# What is the nature of the uranium(iii)–arene bond?†

**DOI:** 10.1039/d3sc04715f

**Published:** 2023-12-14

**Authors:** Sabyasachi Roy Chowdhury, Conrad A. P. Goodwin, Bess Vlaisavljevich

**Affiliations:** a University of South Dakota 414 E Clark St. Vermillion SD 57069 USA bess.vlaisavljevich@usd.edu; b Centre for Radiochemistry Research, The University of Manchester Oxford Road Manchester M13 9PL UK conrad.goodwin@manchester.ac.uk; c Department of Chemistry, The University of Manchester Oxford Road Manchester M13 9PL UK

## Abstract

Complexes of the form [U(η^6^-arene)(BH_4_)_3_] where arene = C_6_H_6_; C_6_H_5_Me; C_6_H_3_-1,3,5-R_3_ (R = Et, iPr, *t*Bu, Ph); C_6_Me_6_; and triphenylene (C_6_H_4_)_3_ were investigated towards an understanding of the nature of the uranium–arene interaction. Density functional theory (DFT) shows the interaction energy reflects the interplay between higher energy electron rich π-systems which drive electrostatic contributions, and lower energy electron poor π-systems which give rise to larger orbital contributions. The interaction is weak in all cases, which is consistent with the picture that emerges from a topological analysis of the electron density where metrics indicative of covalency show limited dependence on the nature of the ligand – the interaction is predominantly electrostatic in nature. Complete active space natural orbital analyses reveal low occupancy U–arene π-bonding interactions dominate in all cases, while δ-bonding interactions are only found with high-symmetry and electron-rich C_6_Me_6_. Finally, both DFT and multireference calculations on a reduced, formally U(ii), congener, [U(C_6_Me_6_)(BH_4_)_3_]^−^, suggests the electronic structure (*S* = 1 or 2), and hence metal oxidation state, of such a species cannot be deduced from structural features such as arene distortion alone. We show that arene geometry strongly depends on the spin-state of the complex, but that in both spin-states the complex is best described as U(iii) with an arene-centred radical.

## Abstract 2

Complexes of the form [U(η^6^-arene)(BH_4_)_3_] where arene = C_6_H_6_; C_6_H_5_Me; C_6_H_3_-1,3,5-R_3_ (R = Et, *i*Pr, *t*Bu, Ph); C_6_Me_6_; and triphenylene (C_6_H_4_)_3_ were investigated towards an understanding of the nature of the uranium–arene interaction.

## Graphical abstract

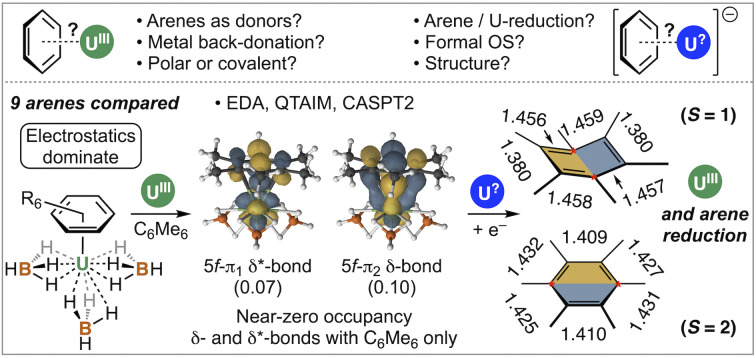

## Introduction

**Fig. 1:**
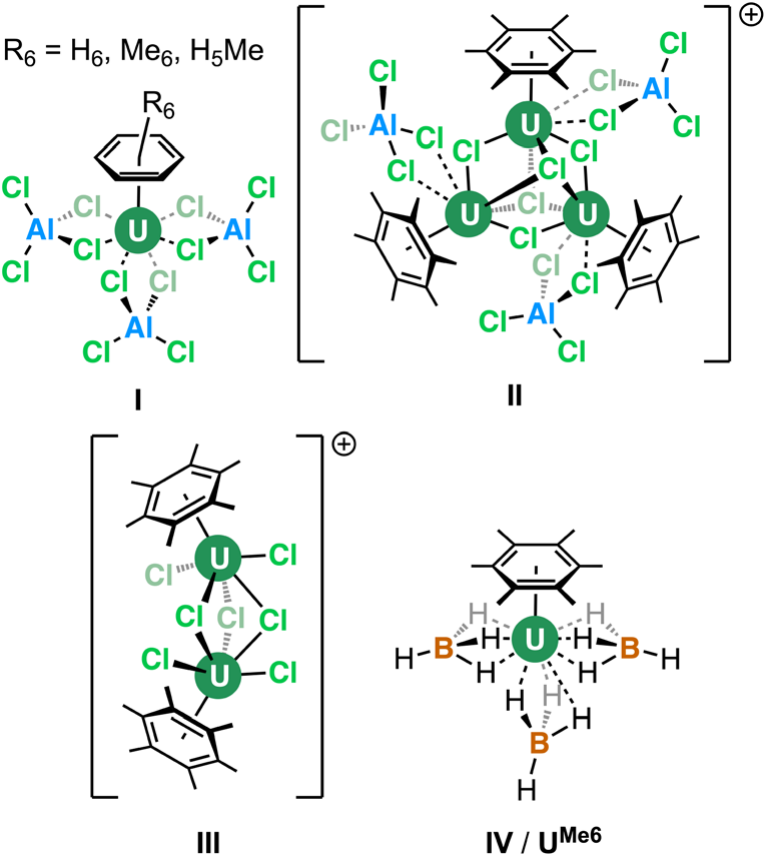
Previously reported “terminal” uranium–arene complexes.

The coordination and organometallic chemistry of uranium has blossomed since the early 1990s.^1^ Despite the wealth of studies into technically challenging syntheses of uranium-element bonds and their attendant experimental and quantum chemical properties,^2^ there is a relative paucity of research into the fundamental nature of “terminal” (neither bridging nor tethered) actinide arene complexes.^3,4^ This is somewhat surprising given the foundational position of complexes such as bis-benzene chromium in our understanding of transition metal chemistry, and its role in chemistry coursework, along with that of “piano-stool” arene complexes in medicine.^5^ Just a handful of simple, terminal uranium–arene complexes have been reported by Cotton, Ephritikhine, Marconi, and Zakharov (I–IV) between 1971 and 1996 which feature examples of both U(iii) and U(iv) (Fig. 1),^6–11^ and this contrasts the wealth of uranium inverse–sandwich complexes which feature bridging arene moieties in varying reduced forms (*i.e.*, C_6_R_6_^*n*−^ where *n* = 2 or 4 commonly).^12,13^

To our knowledge, the bonding in simple terminal uranium arene complexes has not been studied using modern theoretical techniques. Initial reports detailed significant sensitivity to air/moisture, ready displacement of arene ligands by Lewis-basic solvents, and decomposition in the presence of halocarbon solvents, but no description of the nature of the interaction beyond it being “weak”, which contrasts the many air/moisture stable “piano-stool” arene complexes of the later transition metals.^5^ This is despite theoretical studies on bis-benzene actinide complexes,^14–16^ and also matrix isolation experiments with actinide atoms and aromatic molecules.^17^ The growing family of molecules which leverage (tethered) uranium–arene linkages as electron reservoirs to stabilize unusual formal oxidation states,^18–23^ along with recent extension of these works to the transuranium elements neptunium and plutonium,^24^ make a clear case for the development of a modern theoretical description of the uranium–arene interaction to provide insight into future synthetic work and as a starting point for further transuranium research. Herein we have compared variants of [U(arene)(BH_4_)_3_] where arene = C_6_H_6_; C_6_H_5_Me; C_6_H_3_-1,3,5-R_3_ (R = Et, iPr, *t*Bu, Ph); C_6_Me_6_; and triphenylene (C_6_H_4_)_3_ in both terminal and central ring-bound configurations by density functional theory (DFT) and multireference complete active space methods (CASPT2). This has afforded insight into the competing effects of electron richness and energetic matching of the arene fragment to the uranium atom. By exploring the electronic structure of a reduced analogue, [U(C_6_Me_6_)(BH_4_)_3_]^−^, we show that conventional spectroscopic and structural studies of putative U(ii) species may not be conclusive as to the extent of metal- or ligand-based electron population.

## Results and discussion

**Fig. 2:**
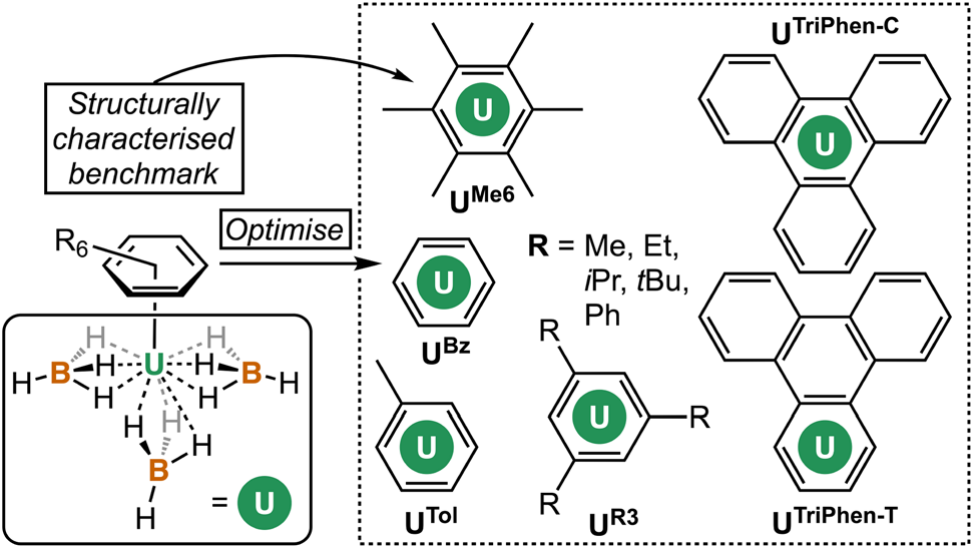
Complexes studied herein.

A range of pure (PBE,^25,26^ TPSS^27^), hybrid (PBE0,^28^ PBE0-D3 ^25,26,29^), and hybrid meta-GGA (TPSSh,^27,30^ TPSSh-D3,^27,29,30^ M06 ^31^) functionals were screened and compared against the X-ray diffraction data from U^Me6^ (see Fig. 2 for key).^10^ Each structure was confirmed to be a minimum by harmonic vibrational analysis. Among the various DFT functionals tested, the PBE0 geometry (U–C mean = 2.903 Å) produced the closest agreement to the experimental mean U–C distances (2.932 Å) for U^Me6^, deviating by only 0.029 Å. However, we note that while the experimental data for U^Me6^ shows two U–C bonds (2.869 and 2.885 Å) slightly shorter than the other four (range 2.947–2.976 Å), all computed geometries gave three shorter U–C distances. The experimental U–B distances (2.482, 2.687, and 2.537 Å) are inequivalent with a mean of 2.568 Å, whereas all calculations gave structures with three equivalent U–B distances. Optimisations were performed in the gas phase, and so the asymmetry observed in the experimental structure plausibly arises from crystal packing effects. The U–B distances using the M06 functional (2.556 Å) gave closest agreement to the experimental mean, but also gave much larger U–C distances than the experimental structure (mean 3.024 Å *vs.* 2.932 Å by experiment), see Table S1 and Fig. S1 for comparisons between the functionals tested.† The PBE0 functional gave a smaller U–B distance (2.525 Å) than experiment, but the deviation along the U–C bond between experiment and theory using PBE0 (0.029 Å) was smaller than that with the M06 functional (0.092 Å).

**Fig. 3:**
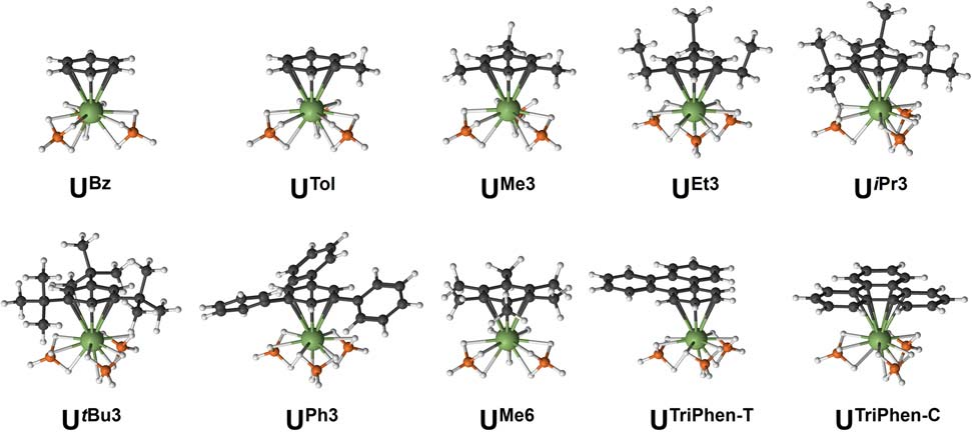
Calculated PBE0 geometries for all complexes studied.

Next, the potential energy surface was explored along the U–C distances in U^Me6^ over a range of ±0.1 Å in 0.05 Å increments. Single-point DFT and CASPT2 calculations were performed at each geometry to obtain the energy difference from the DFT optimised geometry minimum (Fig. S2†). While the energy minimum by CASPT2 was found with U–C distances *ca.* 0.05 Å shorter than the PBE0 geometry minimum (0.073 Å shorter than the experimental structure), the potential energy surface is very shallow (±1.4 kcal mol^−1^ at extremes). When the energy surface along a bond is flat, small energy differences based on method choice can lead to larger differences in the calculated distance. Said another way, the differences in calculated distance are consistent with the accuracy one expects from each method. Since PBE0 shows the smallest deviation in calculated mean U–C distance, the remaining geometry optimisations of compounds in Fig. 2 were carried out using the PBE0 functional, taking the crystal structure of U^Me6^ as the starting point.^10^ See Fig. 3 for the calculated structures of these complexes, and Table 1 for the calculated bond lengths and distances.

**Table 1:** Calculated (PBE0) and experimental mean U–C and U–B distances for complexes studied. Experimental results and the difference from experiment (*Δ*) are included for U^Me6^

Complex	U–C (Å)	U–B (Å)
U^Me6^ (expt.)^10^	2.932	2.568
U^Me6^ (calc.)	2.903 (*Δ* = −0.029)	2.525 (*Δ* = −0.043)
U^Bz^	2.851	2.509
U^Tol^	2.858	2.511
U^Me3^	2.873	2.516
U^Et3^	2.857	2.518
U^iPr3^	2.878	2.519
U*^t^*^Bu3^	2.914	2.522
U^Ph3^	2.888	2.511
U^TriPhen−T^	2.881	2.511
U^TriPhen−C^	3.048	2.514

Within our series, U^Bz^ has the shortest calculated U–C and U–B distances (2.851 Å and 2.509 Å respectively). Greater arene substitution from U^Bz^ → U^Tol^ → U^Me3^ → U^Me6^ leads to an increase in the U–C distances by 0.007, 0.022, and 0.052 Å, respectively, *vs.* that of U^Bz^ (Table 1). Similarly, increasing bulkiness of the arene substituents further lengthens the U–C distances such as going from U^Et3^ (2.857 Å), to U^iPr3^ (2.878 Å), and U*^t^*^Bu3^ (2.914 Å). Both U^Ph3^ (2.888 Å) and U^TriPhen−T^ (2.881 Å) give U–C distances shorter than U^Me6^ and U*^t^*^Bu3^, presumably due to decreased steric clashing with the BH_4_ groups, but both have longer U–C distances than in bulkier U^iPr3^ (2.878 Å; *Δ* = 0.01 Å for U^Ph3^; 0.003 Å for U^TriPhen−T^) which we attribute to electronic factors (*vide infra*). Finally, U^TriPhen−C^ gave the longest U–C distances (3.048 Å) despite having approximately the same steric profile as U^Bz^ (U–C = 2.851 Å), again subsequent analyses suggest this is driven by electronic structure factors.

A topological analysis of the electron density was performed with Bader's Quantum Theory of Atoms in Molecules (QTAIM)^32^ to characterize the U–arene interaction across this series. To contextualise these results, an analysis of the well-known uranocene, [U(COT)_2_],^33,34^ is included at the same level of theory as the neutral arene series. For each neutral arene, three bond critical points (BCPs) were obtained, consistent with the three slightly shorter U–C bond distances. While more detail of this analysis can be found in the ESI,† two parameters are highlighted in Table 2. A large value for the electron density, *ρ*, is indicative of overlap-driven covalency, while the delocalization index, *δ*, is a metric for energy degeneracy-driven covalency – though we are keen to stress that covalency is not a physically observable property and that decomposing it into these categories is not necessarily meaningful. Instead we wish to highlight that these two metrics simply offer insight into the chemical levers which may be pulled to change a metal–ligand interaction (Tables S2–S7† further summarise these data).

**Table 2:** The average electron density, *ρ*, at the bond critical points along the U–C bonds and the average delocalization index, *δ*, of the U–C bonds. All values are expressed in atomic units

Complex^a^	*ρ*	*δ*
[U(COT)_2_]	0.047	0.259
U^Me6^	0.030	0.157
U^Bz^	0.033	0.194
U^Tol^	0.032	0.189
U^Me3^	0.031	0.178
U^Et3^	0.033	0.180
U^iPr3^	0.032	0.173
U*^t^*^Bu3^	0.029	0.159
U^Ph3^	0.031	0.171
U^TriPhen−T^	0.032	0.175

a Note that U^TriPhen−C^ has been excluded as no BCP was observed between the uranium ion and the triphenylene moiety. See Topology analysis and Table S4 in the ESI.

As one might expect, we find that none of the U–C bonds in the U^arene^ series are perfectly covalent, and the same is true of [U(COT)_2_]. The nature of the bond can be characterised by the QTAIM results. At a bond critical point in a perfectly covalent bond, both the Laplacian of the electron density, ∇^2^*ρ*, and the total electronic energy density, *E*(*r*), would be negative. We do not find this to be the case for these systems; instead, we find a positive ∇^2^*ρ* and a negative *E*(*r*) supporting the assignment of a dative bond for all U–C interactions (Table S2†). However, the increased magnitudes of *ρ* and *δ* in [U(COT)_2_] (Table 2) relative to U^arene^ suggests both orbital- and energy degeneracy-driven covalent contributions are greater in [U(COT)_2_], commensurate with its significant chemical stability and previous theoretical descriptions.^33–35^ These differences are not unexpected given one is U(iv), and the other is somewhat softer U(iii), but provide valuable context. For U^arene^, the mean *ρ* and *δ* values remain similar across the series; however, the delocalization index is smaller for U–C bonds when the C-atom has substituents (*i.e.*, mean *δ* values trend U^Me6^ < U^Me3^ < U^Tol^ < U^Bz^) (Table S5†). In this series, both *ρ* and *δ* are small and show limited differences as a function of arene substitution, supporting the characterization of the uranium–arene interaction as predominantly electrostatic.

**Fig. 4:**
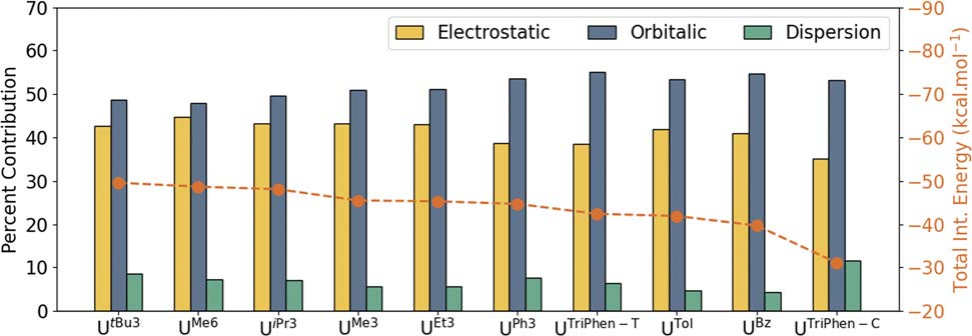
Energy decomposition analysis (PBE0-D3) of the uranium–arene complexes considering the dispersion interaction between the two fragments. Total interaction energies (dotted line) are reported in kcal mol^−1^. The percent contribution of electrostatic, orbital, and dispersion interactions (bar graphs) to the total attractive interaction energy are also shown.

With it shown computationally that the covalent contributions to the U–arene are weak, as suggested by the overall weak interaction observed in the syntheses,^6–11^ an energy decomposition analysis (EDA) was performed to better understand the individual components of the interaction. For EDA, the complexes were considered to be divided into two fragments: U(BH_4_)_3_ and the arene. The total interaction energy (Δ*E*_Int_) was evaluated as a function of electrostatic (Δ*V*_Elstat_), orbital (Δ*E*_Orb_), Pauli repulsion (Δ*E*_Pauli_), and dispersion (Δ*E*_Disp_) contributions. The contributions of individual attractive terms are reported as percentages of the total interaction energy (Fig. 4). A larger percent Δ*V*_Elstat_ contribution implies greater ionic character in the interaction, whereas the percentage of Δ*E*_Orb_ describes the extent of orbital mixing — one possible descriptor of covalent character. See Energy decomposition analysis, Tables S8, and S9 in the ESI.†

In U^Bz^, the total interaction consists of 40.9% electrostatic and 54.8% orbitalic character, and a small contribution (4.3%) from dispersion forces. As the number of methyl groups increases from U^Bz^ → U^Tol^ → U^Me3^ → U^Me6^, the electrostatic contribution gradually increases. This is associated with a gradual increase in the total Δ*E*_Int_; however, the orbital contribution (Δ*E*_Orb_) drops — *i.e.* the interaction becomes stronger with greater methyl group substitution, but also more ionic. Analysis of the frontier orbital energies of the fragments involved by energy decomposition analysis (EDA) suggests that with increasing number of methyl groups at the arene, the energy separation between the frontier orbitals of the two fragments increases (which will decrease mixing—the Δ*E*_Orb_ “covalency” term). In turn, the electrostatic character increases. On the other hand, in the presence of electron-withdrawing (or less electron-donating) substituents on the arene such as in U^Ph3^, the frontier orbitals of the two fragments lie energetically closer to one another, eventually leading to an increase in orbitalic character (Fig. S3†). These results suggest that while the electrostatic (ionic) *vs.* covalent composition of the interaction changes with arene methyl substitution, the main differences in calculated stability of these complexes is derived from electrostatic and dispersion forces across these four.

Significant, albeit unsurprising, contributions from the dispersion forces were found with the heavily alkylated complexes (in order of calculated stability) U*^t^*^Bu3^, U^Me6^, U^iPr3^, while U^Me3^, U^Et3^, and U^Ph3^ were similar to each other and with total stabilities *ca.* 3–4 kcal mol^−1^ lower than the first three (Table S9†). Interestingly, while the Δ*E*_Disp_ contribution for U^Ph3^ (−9.4 kcal mol^−1^) is second only to that of U*^t^*^Bu3^ (−11.0 kcal mol^−1^), the former is calculated to be almost 5 kcal mol^−1^ less stable overall. By all metrics, both terminal and central U^TriPhen^ complexes, U^Tol^, and U^Bz^ are predicted to have very low stability.

Although EDA indicates significant orbitallic interactions between the uranium ion and the arene ligands in all the complexes, further analyses using the energy decomposition analysis-natural orbitals for chemical valence (EDA-NOCV) revealed that the orbital interactions primarily involved reorganisation of the electron density among the f-orbitals on uranium as opposed to transfer between the two fragments which would be expected for a covalent interaction (Fig. S4†). By comparing electron-rich and electron-poor arenes, a picture emerges which intuitively aligns with the idea that the former generates a stronger interaction (thus a more stable complex), while the latter has increased orbital mixing which does not necessarily produce a stronger interaction – certainly not in the case of the complexes examined here. This is reminiscent of discussions surrounding covalency in the mid-actinide elements which often show increased mixing due to energetic lowering of the 5f orbitals, but which don't necessarily show increased markers of covalency by other computational methods.^36–42^ Of course, such a separation of the concept of “covalency” into two different categories is somewhat academic, and instead, these data simply serve to describe how one might engineer ligands to produce a more stable U(iii) arene adduct.

**Fig. 5:**
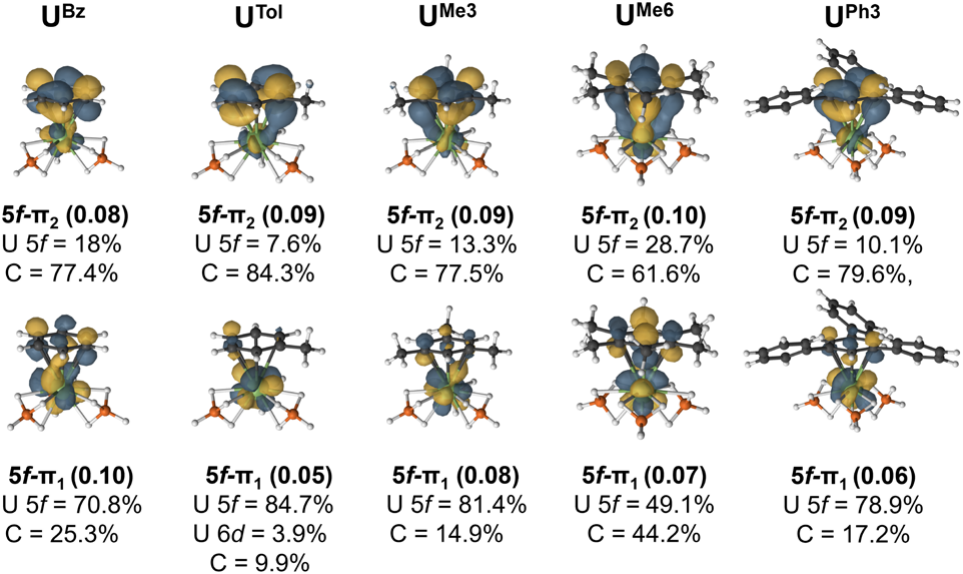
The CASSCF active natural orbitals with dominant mixing between the metal 5f and the arene ligand π orbitals (5f–π_1_ and 5f–π_2_) of selected complexes studied. An isovalue of 0.04 a.u. is used. All active space orbitals are shown in Fig. S8–S20.†

The extent of overlap between the uranium 5f and arene-π orbitals within a (9*e*, 13*o*) active space was evaluated for all complexes (see Fig. S8–S20†) except for the two U^TriPhen^ congeners which were omitted due to their lower stability and larger computational cost. In all studied molecules, at least two molecular orbitals show notable overlap (of U 5f–ligand π_*n*_ parentage; *n* = 1 or 2 to denote the two orbitals) commensurate with a *δ*/δ* bond-pair – albeit in the neutral species these orbitals are essentially unoccupied (*vide infra* for charged species). In each pair, one orbital is predominantly 5f-character (5f–π_1_), and the other is mostly arene π-character (5f–π_2_). Fig. 5 shows the CASSCF natural orbitals with dominant mixing between the metal 5f, and the arene ligand π orbitals for selected species (see Fig. S5† for all species). We wish to highlight to the reader that in this methodology there are no associated energies with these natural orbitals, only occupations numbers, and so the numbering scheme used is somewhat arbitrary, and chosen for internal consistency and ease of comparison. In U^Bz^, the 5f–π_1_ orbital consists of 70.8% U and 25.3% arene character, while the corresponding 5f–π_2_ orbital shows 18% 5f and 77.4% arene character. Introduction of a methyl group in U^Tol^ gave a slightly greater Δ*E*_Int_ (*vide supra*); however, the reduction in molecular symmetry also decreases overlap between the 5f and π orbitals. In some cases, U 6d orbitals also contribute (*ca.* 2–4%), although to a lesser degree than the 5f, (see Fig. S8–20† for a more detailed description). to previous observations that 5f involvement with the early actinides can be the most significant driver of bonding differences,^43–47^ though this strongly depends on both the actinide-ion and the donor.^36–42^

By comparing U^Tol^, U^Me3^, and U^Me6^, we see the influence of arene symmetry on orbital composition. In U^Tol^, the 5f–π_1_ (84.7% 5f; 3.9% 6d; 9.9% C – total 88.6% U) and 5f–π_2_ (7.6% 5f, 84.3% C) orbitals are strongly polarised; whereas in U^Me3^, the corresponding 5f–π_1_ (81.4% 5f; 14.9% C) and 5f–π_2_ (13.3% 5f, 77.5% C) orbitals are somewhat less polarised. Then, in U^Me6^, the polarity of these two orbitals is reduced significantly such that 5f–π_1_ (49.1% 5f; 44.2% C) is a fairly even split between 5f and arene-π contributions, and while 5f–π_2_ (28.7% 5f; 61.6% C) is somewhat dominated by arene π contribution, it is much less polarised than in U^Tol^, U^Me3^. Complex U^Me6^ is also the only one where we see a clear *δ* (5f–π_2_) and δ* (5f–π_1_) interactions, despite it having mean U–C distances similar to those of U*^t^*^Bu3^ and U^Ph3^, which do not show these interactions (Fig. 5). We attribute this unique aspect of U^Me6^ to the higher symmetry of the arene ligand. With the exception of U^Bz^ and U^Et3^ (which also have the shortest calculated U–C distances) the sum of arene content in U–dominant 5f–π_1_ orbital, and U-content in the arene-dominant 5f–π_2_ orbital (see Fig. S5†), perfectly matches the trend of Δ*E*_Int_ from the EDA calculations – suggesting that the more mixed (less polar) these two orbitals are, the greater the calculated stability of the complex *vs.* its constituents. Though we note that in all cases the occupancy of these two orbitals is exceptionally low (*ca.* 0.05–0.10) and therefore no bond, in a chemically intuitive sense, is present.

Across all eight complexes, there is no clear trend in the role of U–C distances and the degree of mixing. Indeed, U^Me6^ shows the second longest distances and by far the greatest mixing (second most negative Δ*E*_Int_), while U*^t^*^Bu3^ (most negative Δ*E*_Int_) has U–C distances only slightly longer and is essentially as polarised as all other examples. The greater stability of the latter is driven by dispersion interactions. To examine the influence of U–C distance on mixing, we have analysed the CASSCF natural orbitals of partially optimised U^Me6^ complexes obtained by varying the mean U–C distances by ±0.10 Å in 0.05 Å increments (Fig. S6†). At the shortest (−0.10 Å, representing a *ca.* shortening *vs.* the experimental structure), we find two additional mixed 5f–π orbitals, namely 5f–π_3_ and 5f–π_4_, which are comprised of >80% arene, and U 5f character, respectively. Despite the changes observed in the metal–ligand interaction while compressing the uranium–arene distance, the CASPT2 energy surface is extremely flat (*vide supra*) (Fig. S2†).

**Fig. 6:**
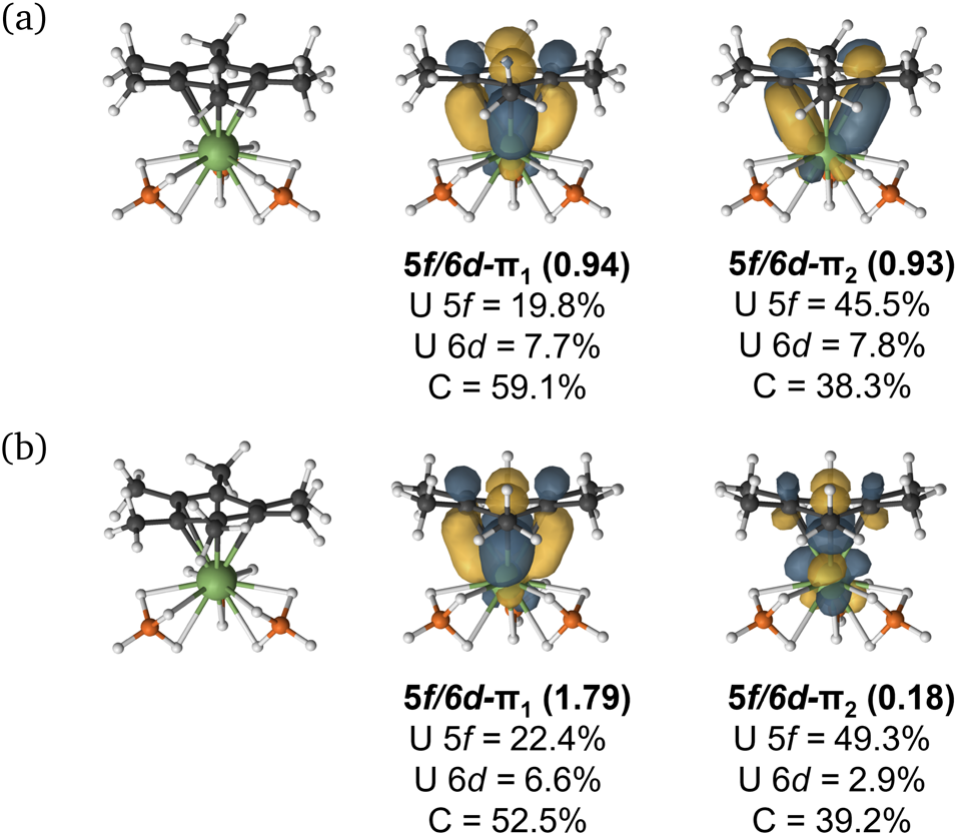
(a) High-spin (*S* = 2) and (b) intermediate-spin (*S* = 1) PBE0 molecular geometries and selected CASSCF active natural orbitals of [U^Me6^]^−^ using a (10e, 18o) active space with dominant mixing between the metal 5f, 6d and the arene ligand π orbitals. An isovalue of 0.04 a.u. was used.

Given the dependence upon electron-richness of the arene in directing the U–arene interaction energy in these complexes, we have sought to explore the impact of adding an electron to form [U^Me6^]^−^ (Fig. 6) and to see whether this would enhance back-donation to the arene ring *via* increased population of U-centered electrons, or whether the arene would bear the majority of the charge, strengthening the interaction *via* the same mechanism described above. The parent species U^Me6^ is well described as U(iii) with a C_6_Me_6_ ring electrostatically bound to it. The addition of another electron to form [U^Me6^]^−^ can give both high-spin (HS; *S* = 2) and intermediate-spin (IS; *S* = 1) states; therefore, we have fully optimised the molecular geometries of both species with DFT and confirmed both are minima by harmonic vibrational analysis. The calculated mean U–C distances in [U^Me6^]^−^ (HS = 2.709 Å; IS = 2.703 Å) are contracted by *ca*. 0.2 Å relative to neutral parent U^Me6^ (2.903 Å). At this stage, we highlight for the reader that this decrease in U–C upon reduction from U(iii) to formal U(ii) is somewhat larger than that seen in a range of recently reported low oxidation state U–arene complexes.^18–23^ For example, in Meyer's formally divalent [U{mes(OAr^Ad,Me^)_3_}]^−^ complex, the U–C range is 2.597–2.633 Å, while in the trivalent precursor it is 2.729–2.774 Å, a contraction of roughly 0.10–0.15 Å.^18^ Similarly, in trivalent [U(NHAr^iPr^_6_)_2_]^+^, the U–C range is 2.828–3.059 Å, while in the neutral divalent species [U(NHAr^iPr^_6_)_2_] it is 2.723–2.902 Å—again, *ca.* 0.10 Å contraction upon reduction.^19^ Nevertheless, as a U(ii) complex with a terminal U–{C_6_R_6_} linkage has not been isolated to date, we don't wish to speculate on what influence the tethering groups in the reported complexes have upon the final U–C distances, and thus whether the contraction seen in our example is realistic for this complex, or not. That said, the relative energies of the orbitals provide some insight into the reactivity of the complex. If we consider the CASSCF canonical orbitals and their respective energies, the metal dominated orbitals are well-separated from the ligand-dominated orbitals suggesting an increased reactivity (Fig. S7 and Table S10†). This supports that the reduced complex will be, at best, challenging to isolate.

**Fig. 7:**
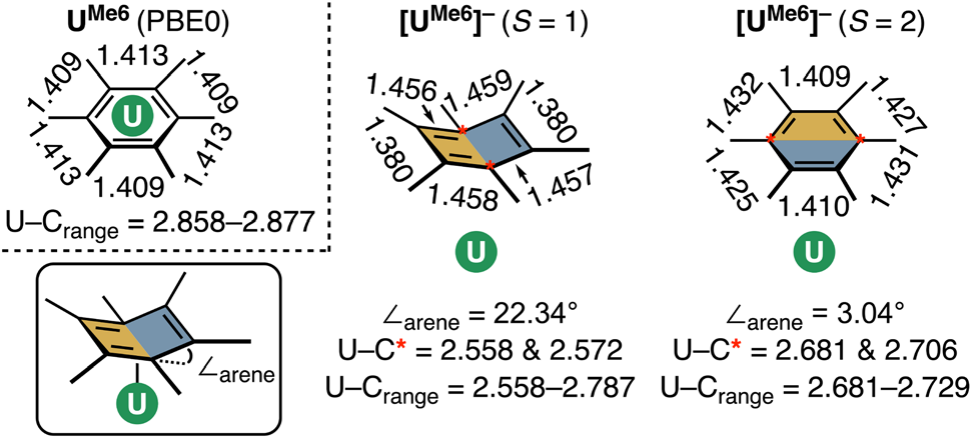
A schematic of the optimised U–arene geometry in neutral [U^Me6^], and both intermediate-spin (*S* = 1) and high-spin (*S* = 2) [U^Me6^]^−^, highlighting U–C and C–C bond lengths (Å), and the *∠*_arene_ parameter.

While both HS and IS geometries of [U^Me6^]^−^ exhibit similar mean U–C distances, a notable deformation is apparent in the arene ring in the *S* = 1 (IS) state which resembles an “open book” conformation (*∠*_arene_ = 22.34° – see Fig. 7 for an illustration of this deformation), and this seems to facilitate a stronger *δ* – bonding interaction. Such deformation is characteristic of Jahn–Teller distortion of benzene rings.^48,49^ Furthermore, the C–C bonds in this spin-state show a pattern of four long (mean: 1.457 Å) and two short (mean: 1.379 Å) distances, which compares reasonably well to the pattern in [U(NHAr^iPr^_6_)_2_] (four long, mean: 1.425 Å, two short, mean: 1.396 Å), noting that the *∠*_arene_ in this complex is only 9.3(2)°, and so one would expect a lesser degree of disruption to the aromatic system.^19^ A similar deformation is seen in other complexes.^20,22,23^ Conversely, in the *S* = 2 (HS) state of [U^Me6^]^−^ the arene geometry remains planar with no tell-tale structural signs of arene reduction, much like in [U{mes(OAr^Ad,Me^)_3_}]^−^ and [U{C_6_H_3_-1,3,5-(C_6_H_4_-2-NAd)_3_}]^−^.^18,21^ While this planar (HS) *vs.* distorted (IS) observation could be an artefact of our calculations, as noted above, complexes possessing U–arene linkages with both of these geometries have been reported.^18–23^ Indeed, Meyer and co-workers calculated both HS and IS spin-states of their [U{mes(OAr^Ad,Me^)_3_}]^−^ anion and found the same relationship between spin-state and arene distortion.^18^

CASPT2 calculations with an active space of (10e, 18o) were performed on the DFT-optimised geometries for both spin states. These data show that the *S* = 2 (HS, planar arene ring) structure is more stable than the *S* = 1 (IS, folded-book arene ring) geometry by 2.8 kcal mol^−1^, which is consistent with the DFT energy difference of 1.5 kcal mol^−1^. Though we note that these energy differences are within the realm of crystal packing forces in molecular systems.^50^ Analysis of CASSCF natural orbitals for both the HS and IS states of [U^Me6^]^−^ reveals that out of ten electrons considered in the active space, six remain located in the three π-orbitals of the arene, two occupy 5f orbitals on the metal ion, and the remaining two are in *δ* – bonding (5f/6d–π_*n*_) orbitals dominated by either metal 5f or ligand π orbitals, with some 6d participation. Therefore, in both spin-states, the total metal composition approaches three electrons rather than the four which would indicate metal-based reduction (Fig. 6). In the HS configuration, *δ* – bonding orbitals have occupation numbers of 0.94 and 0.93, respectively, with fairly polar compositions – the 5f/6d–π_1_ is comprised of 19.8% 5f, 7.7% 6d (total 27.5% U), 59.1% arene-C contributions, and the 5f/6d–π_2_ is 45.5% 5f, 7.8% 6d (total 53.3% U), 38.3% arene–C. In the IS configuration, the *δ* – bonding orbital (5f/6d–π_1_) has an occupation number of 1.79 with a highly delocalised composition (22.4% 5f, 6.6% 6d (total 29% U) 52.5% arene–C), and the 5f/6d–π_2_, which is δ* – anti-bonding in this spin-state, has an occupation number of 0.18, and is similarly delocalised across U 5f and arene–C orbitals (49.3% 5f, 2.9% 6d (total 52.2% U), 39.2% arene–C).

One can consider the difference in orbital occupation numbers between U^Me6^ and [U^Me6^]^−^ to help assign whether the electron added upon reduction is metal- or arene-based. In the IS spin-state two electrons reside in U 5f orbitals in addition to the six arene π-electrons, and the remaining two are distributed over the *δ* (occupation number 1.79) and δ* (occupation number 0.18) orbitals (Fig. 6), though the composition of these only sums to approximately one additional metal-based electron. The same is true for the HS spin state. Thus, we find that the *δ* bonding in [U^Me6^]^−^ is stronger than that of U^Me6^—one would expect this from the discussion of electrostatic binding in the neutral parent complexes above as upon reduction the arene ring takes on a greater negative charge, and thus binds more tightly. Furthermore, in the IS spin-state this additional arene electron population causes the distortion we observe which is similar to that shown in some reported complexes,^19,20,22,23^ while in the HS spin-state the arene remains planar as shown in some other reported complexes.^18,21^ In the system studied herein, we note that (i) in both spin-states the arene orbital occupation is greater than that of a neutral arene by approximately one electron, and (ii) the percentage occupation of orbitals with U-parentage, including the two essentially pure 5f orbitals, is only ever approximately three—not four as would be expected for metal-based reduction from U(iii) to U(ii).

**Fig. 8:**
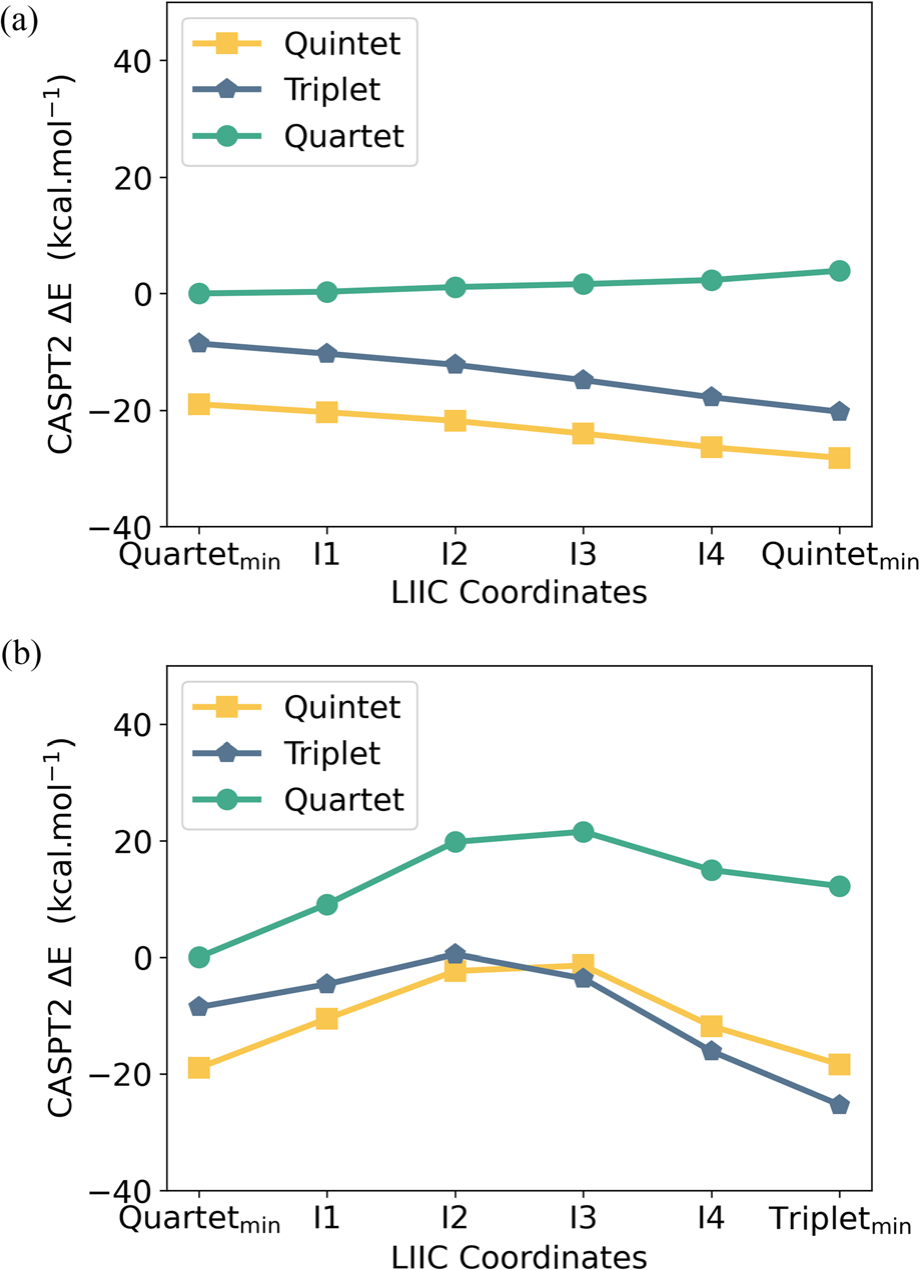
CASPT2 computed potential energy surface using an (*ne*, 18*o*) active space along the linear interpolation of internal coordinates (LIIC) between the PBE0 optimized (a) U^Me6^ high spin (*S* = 3/2) and [U^Me6^]^−^ high spin (*S* = 2) geometries and (b) U^Me6^ high spin (*S* = 3/2) and [U^Me6^]^−^ intermediate spin (*S* = 1) geometries. Note that the *S* = 2 state on its DFT-optimised structure, right most point in (a), is the lowest energy point in either plot.

To better understand the accessibility and stability of either spin state of [U^Me6^]^−^, we analysed the potential energy surface (PES) between the U^Me6^ and [U^Me6^]^−^ optimised geometries by evaluating the linearly interpolated internal coordinates (LIIC) followed by CASPT2 calculations on these intermediate geometries (see Fig. 8). The CASPT2 PES reveals that upon reduction of U^Me6^ to [U^Me6^]^−^ (*i.e.*, while in the U^Me6^ geometry), the *S* = 2 spin state is favored by 10.4 kcal mol^−1^ (Fig. 8a). Recall that upon geometric distortion, the *S* = 1 (IS, distorted arene ring) state is slightly less stable than the *S* = 2 (HS, planar arene ring) state. Moreover, there is an activation barrier of 9.0 kcal mol^−1^ associated with reaching the IS ground state geometry (Fig. 8b). Presumably this is related to the greater degree of arene structural reorganisation *vs.* that of the HS state where no barrier is observed.

Mulliken spin population analyses on the U- and arene–C atoms reveals that in the parent trivalent U^Me6^ complex, the metal ion solely bears the unpaired spin (2.94, close to the theoretical value 3). However, in both spin-states of [U^Me6^]^−^ significant spin accumulation is reported on the arene. In the HS state of [U^Me6^]^−^, a putative ferromagnetic interaction between the uranium and the arene is found, with spin populations of 3.07 and 0.86 respectively. This arrangement could be interpreted as the three U 5f electrons ferromagnetically coupling with an arene radical. Spin-pairing produces significant spin-delocalization in the IS state of [U^Me6^]^−^, where instead of three U 5f electrons anti-ferromagnetic coupled to an arene-centred radical, we find a spin-population of only 2.21 on U, and the arene having a spin population of −0.21. Therefore, the IS state of [U^Me6^]^−^ appears to be more delocalised than its high-spin counterpart. This is also reflected in U and arene partial charges (Tables S11 and S12†) where the addition of an electron does not impact the partial positive charge on the metal ion; however, the cumulative partial negative charge on the arene carbon atoms increases, consistent with the assignment of arene reduction.

## Conclusions

A range of neutral U(iii)-arene adducts of the form [U(η^6^-arene)(BH_4_)_3_] where arene = C_6_H_6_; C_6_H_5_Me; C_6_H_3_-1,3,5-R_3_ (R = Et, iPr, *t*Bu, Ph); C_6_Me_6_; and triphenylene (C_6_H_4_)_3_ were studied towards a better understanding of the bonding interaction in such complexes. By way of an energy decomposition analysis at the DFT level of theory we find that the complex with C_6_Me_6_ is slightly less stable than the C_6_H_3_-1,3,5-*t*Bu_3_ complex, and closely approached by the C_6_H_3_-1,3,5-iPr_3_ complex, with these differences driven primarily by slightly greater dispersion interactions in the latter two. Though we are keen to note these differences are within the error of calculation, and also of the same scale as crystal packing forces in molecular systems. Given that [U^Me6^] is an isolatable crystalline complex, it may be that these other two are sensible targets for future synthetic investigation. We find a positive correlation between arene electron richness and total (negative) interaction energy in all complexes studied, with orbitallic (energy matching) and dispersion forces playing a smaller role in determining the order of stability. By comparing the electron density and delocalisation index in these complexes with that of [U(COT)_2_] we find that all [U(η^6^-arene)(BH_4_)_3_] complexes have a U–C interaction which is “less covalent” than the former by a significant degree, but all U^arene^ complexes are roughly equivalent to each other. Analyses of the CASSCF active orbitals (except for the triphenylene systems which were omitted) for these complexes reveals in all cases that a combination of two 5f/6d–π_*n*_ (*n* = 1 or 2) mixed very low occupancy orbitals (*ca*. 0.05–0.10 electrons) describes the bonding interaction, and below these sits the three essentially pure 5f orbitals. Taken together, these results show that the bonding in the U–arene fragment is best described as electrostatic in nature whereby a well-localised U(iii) cation binds electron-rich arene rings mostly due to simple electrostatics, but also due to a very minor degree of back-donation forming very low occupancy 5f–π_*n*_ orbitals. Interestingly, there is a small dependence upon arene symmetry in these interactions, with C_6_Me_6_ being the only arene to show *δ* – bonding interactions. In this example, bringing the arene into closer proximity to the metal (by 0.10 Å) revealed additional 5f–π_*n*_-based *δ* – bonding interactions. Irrespective of arene symmetry or identity, the degree of U-6d interaction is minimal in all U(iii) complexes examined. Symmetry-allowed combinations of (occupied) 5f and arene *ψ*_1–3_ orbitals can form bonding interactions, as can the (unoccupied) 6d with these same arene orbitals. The former likely has a greater stabilising effect in the case of U(iii), though not for U(ii) where substantial back-bonding is seen (*vide infra*).

By exploring the electronic structure of a putative U(ii) complex, [U^Me6^]^−^, by DFT and multireference (CASSCF) calculations we have afforded insight into the growing class of low oxidation state uranium-complexes which feature U–arene interactions. We find that the arene geometry in formally U(ii) complexes with U–arene interactions is an indication of the overall spin state (Fig. 6), but that geometric parameters cannot be used to describe the degree of arene, and hence uranium, electron localisation in complexes such as these. The high-spin state (*S* = 2) of [U^Me6^]^−^ (which features a planar arene ring), is best described as U(iii) ferromagnetically coupled to an arene radical. In the case of the low spin (*S* = 1) congener (which features a somewhat “open book” shaped arene ring), this is also best described as U(iii) with substantial delocalisation into an anti-ferromagnetically coupled arene ring. These descriptions are derived both from (i) CASSCF natural orbital composition, (ii) partial charge analysis, and (iii) spin population analysis. Given that in both spin states of [U^Me6^]^−^ we find that the arene electron population is essentially one greater than that of a neutral benzene ring (*i.e.*, seven rather than six) it is difficult to avoid comparisons between more classical complexes which feature unequivocal mono-anionic arene rings such as ferrocene. The molecular orbital diagram of ferrocene shows that six electrons (of the total 3d^6^ in Fe^0^) reside in non-bonding metal-based orbitals,^51^ while the remaining two form a bonding interaction with the Cp ligands (*a*_1g_ in *D*_5d_ symmetry), yielding a formal oxidation state of Fe(ii) in this case. By way of analogy for both spin-states here we find two essentially pure (non-bonding) 5f orbitals, and a combination of two mixed *δ* – bonding orbitals which sum to at least one arene-based electron, and almost one metal-based electron. By the same logic which describes the Fe-centre of ferrocene as Fe(ii), the U-centre in [U^Me6^]^−^ is U(iii) as the electron count at U is three – the only source of ambiguity comes from whether one regards the arene ring as an anion in systems such as this, which it is in this example. Given that both *S* = 1 and *S* = 2 spin-states of [U^Me6^]^−^ would be expected to be silent by conventional X-band EPR spectroscopy which is usually the work-horse frequency for such chemistry, and both would be expected to give very similar magnetometry traces (certainly the dearth of formal U(ii) complexes means that a baseline cannot be adequately defined as of yet), we tentatively suggest that the combination of X-ray diffraction data and these two techniques would not be sufficient to describe the true oxidation state of uranium in [U^Me6^]^−^. Indeed, this argument may extend to any formal U(ii) complexes which bear U–arene interactions to arenes with low-lying unoccupied orbitals. Future investigations in this area will benefit immensely from alternative spectroscopic techniques such as X-ray absorption or emission spectroscopies. Of course, oxidation state is both a formalism and a continuum, but we hope that by understanding the degree of metal- or ligand-based reduction in such systems, we can arrive at a better rationalisation of their physicochemical properties.

## Computational details

The geometry optimizations of all the complexes were carried out using density functional theory (DFT) starting from the crystal structure of the U^Me6^ complex as the initial geometry.^10^ Two pure DFT functionals PBE,^25,26^ and TPSS,^27^ one hybrid functional PBE0,^28^ and two hybrid meta-GGA functionals TPSSh^27,30^ and M06 ^31^ were employed. To investigate the impact of dispersion on the molecular geometries, the structures were also computed by employing the PBE0-D3 and TPSSh-D3 functionals which include Grimme's D3 correction^29^ with the original damping function. The uranium was treated with def-TZVP basis set and corresponding ECP,^52,53^ while the def2-TZVP basis set^54^ was employed for the rest of the elements. For each geometry optimization, convergence threshold to the Cartesian gradient was set to 1 × 10^−4^ a.u. All the geometries were confirmed as minima by means of harmonic vibrational analysis. The resolution of identity (RI) approximation was also employed for integral evaluation.^55^ These DFT calculations were performed with the Turbomole program package V7.3.^56^

To characterize the bonding between the uranium and arene moieties in the previously described DFT computations, topological analysis of the electron density was performed with the Bader's Quantum Theory of Atoms in Molecules (QTAIM)^32^ as implemented in the Multiwfn 3.8 program.^57^ The bonding was further characterized by performing the energy decomposition analysis as implemented in the Amsterdam Density Functional program^58^ using the hybrid PBE0 functional.^28^ The TZP basis set was used on all the atoms and similar to the geometry optimization, Grimme's D3 dispersion correction was included. The relativistic corrections were taken into account using the scalar relativistic zero-order regular approximation (ZORA).^59^ No core electrons were frozen.

For the wavefunction based analysis, the DFT optimized geometries were subjected to the multiconfigurational complete active space (CASSCF)^60^ calculations followed by second-order perturbation theory (CASPT2).^61,62^ In the CASSCF calculations, an active space of 9 electrons in 13 orbitals (9e, 13o) was used for the neutral complexes. This includes three f electrons of the uranium ion distributed in the seven 5f orbitals, and six π electrons of arene in the six π orbitals. To test the importance of the size of the actives space, calculations with a larger active space including the 6d shell were performed for both U^Me6^ with (9*e*, 18*o*) and for [U^Me6^]^−^ with (10*e*, 18*o*). The energies and occupation numbers were minimally impacted for the U^Me6^ complex (Tables S13–S15 and Fig. S15–S20†). However, for the *S* = 2 state of [U^Me6^]^−^ the contribution of the 5f atomic orbitals to the natural orbitals changed significantly and the energy profile was stabilized. Therefore, the larger active space is employed for discussions of [U^Me6^]^−^ species (*vida infra*).

In the CASPT2 calculations the standard definition of the zero-order Hamiltonian (IPEA = 0.25 a.u) is used.^63^ To exclude the possible intruder states an imaginary shift of 0.2 a.u is applied. The scalar relativistic effects were included at the CASSCF/CASPT2 level using the second order Douglas–Kroll–Hess Hamiltonian^64^ and relativistic all electron ANO-RCC basis sets.^65^ We use [9s,8p,6d,4f,2g,1h] contraction for uranium, [4s,3p,2d,1f] for the boron, and carbon atoms of the first coordination sphere, [3 *s*,2*p*,1*d*] for the peripheral carbons, and [1 *s*] for hydrogen atoms. Cholesky decomposition^66^ in conjunction with local-exchange screening was used to reduce the computational cost. All the CASSCF and CASPT2 calculations are carried out using OpenMolcas^67^ software suite.

## Data availability

Cartesian coordinates for all optimized structures and intermediates along the LIIC are included as ESI.† To ensure reproducibility, the input and output files associated all calculations are available both in a FigShare repository (https://doi.org/10.6084/m9.figshare.24759789) and in an ioChem-BD repository (https://doi.org/10.19061/iochem-bd-6-321).

## Author contributions

CAPG and BV conceived the original concept and directed the investigation. SRC and BV performed all calculations used in the manuscript, and SRC was supervised by BV. CAPG wrote the first draft of the introduction and conclusions sections, and SRC wrote the first draft of the results, with further iterations of all sections conducted jointly between all authors.

## Conflicts of interest

There are no conflicts to declare.

## Supplementary Material

SC-015-D3SC04715F-s001

SC-015-D3SC04715F-s002
