# Supplementary material for: What is the nature of the uranium(iii)–arene bond?
Source: Chem Sci. 2023 Dec 14;15(5):1810–9. doi: 10.1039/d3sc04715f (PMC10829017; doi:10.1039/d3sc04715f)
Supplement: SC-015-D3SC04715F-s002 [file SC-015-D3SC04715F-s002.pdf]

Electronic Supplementary Material (ESI) for Chemical Science.
This journal is © The Royal Society of Chemistry 2023

Supporting Information for: What is the nature of the uranium(III)-arene bond?

Sabyasachi Roy Chowdhury,† Conrad A. P. Goodwin,*,‡ and Bess Vlaisavljevich*,†

†*Department of Chemistry, University of South Dakota, Vermillion SD, 57069, USA*

‡*Centre for Radiochemistry Research, The University of Manchester, Oxford Road, Manchester, M13 9PL UK. Department of Chemistry, The University of Manchester, Oxford Road, Manchester, M13 9PL UK*

E-mail: conrad.goodwin@manchester.ac.uk; bess.vlaisavljevich@usd.edu

List of Tables

S1 Comparison of the functional dependence of computed U−C and U−B bond distances in Å for the $\mathbf{U^{Me_6}}$ complex with respect to experiment.[1] S6

S2 The average topological properties of U−C bonds computed at the bond critical points (BCPs). All values are expressed in atomic units. S9

S3 The average topological properties of U−B bonds of the U^{Me_6} complex, computed at the bond critical points (BCPs). All values are expressed in atomic units. S9

S4 The PBE0-D3 average U−C distance of the $U^{TriPhen-C}$ complex together with the average topological properties of the U−C bonds computed at the bond critical points (BCPs). All values are expressed in atomic units. S9

S5 Delocalization indices (δ) between the uranium and arene carbon atoms of all the $\mathbf{U^{arene}}$ complexes. S9

S6 Delocalization indices (δ) between the uranium and boron atoms of all the $\mathbf{U}^{\mathbf{arene}}$ complexes. S10
S7 Delocalization indices (δ) between uranium and the carbon atoms of the $[U(COT)_2]$ complex. S10
S8 Energy decomposition analysis of the uranium-arene complexes. All energies are reported in kcal/mol. The percent contribution of electrostatic and orbitallic interactions to the total attractive interaction energy is given in the parentheses. S12
S9 Energy decomposition analysis of the uranium-arene complexes considering the dispersion interaction between the two fragments. All energies are reported in kcal/mol. The percent contribution of electrostatic, orbital, and dispersion interactions to the total attractive interaction energy is given in the parenthesis. S12
S10 Canonical orbital energies (in a.u.) from the 18 orbital active space calculations. . . S18
S11 Mulliken spin population analysis from CASSCF-(9*e*,18*o*) for the high-spin quartet state of $\mathbf{U}^{\mathbf{Me_6}}$, and with (10*e*,18*o*) active space for the high-spin quintet state of $[\mathbf{U}^{\mathbf{Me_6}}]^-$, and the intermediate-spin triplet state of $[\mathbf{U}^{\mathbf{Me_6}}]^-$. S18
S12 LoProp charge analysis from CASSCF-(9*e*,18*o*) for the high-spin quartet state of $\mathbf{U}^{\mathbf{Me_6}}$, and with (10*e*,18*o*) active space for the high-spin quintet state of $[\mathbf{U}^{\mathbf{Me_6}}]^-$, and the intermediate-spin triplet state of $[\mathbf{U}^{\mathbf{Me_6}}]^-$. S18
S13 Absolute CASPT2 (9*e*,13*o*) and PBE0 energies (a.u.) of the ground quartet state of the $\mathbf{U}^{\mathbf{Me_6}}$ complex corresponding to the PES of Figure S2. . . . . S19
S14 Absolute CASPT2 energies (a.u.) corresponding to the PES of Figure 8a. An Active space of (9*e*,18*o*) was used for evaluating E_{Quartet}, and the E_{Quintet}, and E_{Triplet} were computed using (10*e*,18*o*) active space. S19
S15 Absolute CASPT2 energies (a.u.) corresponding to the PES of Figure 8b. An Active space of (9*e*,18*o*) was used for evaluating E_{Quartet}, and the E_{Quintet}, and E_{Triplet} were computed using (10*e*,18*o*) active space. S19

List of Figures

S1 Comparison of the functional dependence of computed U−C and U−B bond distances in Å for the geometries of the $\mathbf{U^{Me_6}}$ complex with respect to experiment.[1] . S6

S2 Potential energy surface in kcal/mol of the $\mathbf{U^{Me_6}}$ complex, computed with PBE0 and CASPT2, along the average U−C bond distance. The relative energy (ΔE) is given with reference to the energy on the PBE0 optimized geometry computed with each of the methods. S7

S3 Frontier orbital energies in kcal/mol of the two fragments and the total complex for (a) $\mathbf{U^{Bz}}$, (b) $\mathbf{U^{Tol}}$, (c) $\mathbf{U^{Me_3}}$, (d) $\mathbf{U^{Me_6}}$, and (e) $\mathbf{U^{Ph_3}}$. S14

S4 The ETS-NOCV deformation density of the $\mathbf{U^{Ph_3}}$ with isosurface value of 0.01 a.u., as recommended in the ADF manual. The associated $\alpha - \Delta\rho$ ΔE = -10.465 kcal/mol. No surface is visible at the lower $\alpha - \Delta\rho$ ΔE or $\beta - \Delta\rho$ ΔE values. S15

S5 CASSCF active natural orbitals with dominant mixing between the metal $5f$ and the ligand π orbitals (labeled $5f$-π_n) of the eight complexes are shown. The full active space orbitals are shown in the Figures S8 to S20. An isovalue of 0.04 a.u is used. S16

S6 The CASSCF active natural orbitals of the $\mathbf{U^{Me_6}}$ complex with significant metal-ligand overlap are shown with respect to the average uranium-arene distances. The deviation from the equilibrium uranium-arene distances in Å are reported underneath of the orbitals. S17

S7 Canonical orbital energy diagram from the 18 orbital active space calculations. . . S17

S8 The CASSCF active natural orbitals of the $\mathbf{U^{Bz}}$ complex from the ($9e$, $13o$) active space. An isosurface value of 0.04 a.u. was used. Occupation numbers and atomic contributions to the orbitals are included. S20

S9 The CASSCF active natural orbitals of the $\mathbf{U^{Tol}}$ complex from the ($9e$, $13o$) active space. An isosurface value of 0.04 a.u. was used. Occupation numbers and atomic contributions to the orbitals are included. S21

S10 The CASSCF active natural orbitals of the $\mathbf{U^{Me_3}}$ complex from the (9*e*, 13*o*) active space. An isosurface value of 0.04 a.u. was used. S21

S11 The CASSCF active natural orbitals of the $\mathbf{U^{Et_3}}$ complex from the (9*e*, 13*o*) active space. An isosurface value of 0.04 a.u. was used. Occupation numbers and atomic contributions to the orbitals are included. S22

S12 The CASSCF active natural orbitals of the $\mathbf{U^{iPr_3}}$ complex from the (9*e*, 13*o*) active space. An isosurface value of 0.04 a.u. was used. Occupation numbers and atomic contributions to the orbitals are included. S22

S13 The CASSCF active natural orbitals of the $\mathbf{U^{tBu_3}}$ complex from the (9*e*, 13*o*) active space. An isosurface value of 0.04 a.u. was used. Occupation numbers and atomic contributions to the orbitals are included. S23

S14 The CASSCF active natural orbitals of the $\mathbf{U^{Ph_3}}$ complex from the (9*e*, 13*o*) active space. An isosurface value of 0.04 a.u. was used. Occupation numbers and atomic contributions to the orbitals are included. S23

S15 The CASSCF active natural orbitals of the $\mathbf{U^{Me_6}}$ complex from the (9*e*, 13*o*) active space. An isosurface value of 0.04 a.u. was used. Occupation numbers and atomic contributions to the orbitals are included. S24

S16 The CASSCF active natural orbitals of the $\mathbf{U^{Me_6}}$ complex from the (9*e*, 18*o*) active space. An isosurface value of 0.04 a.u. was used. Occupation numbers and atomic contributions to the orbitals are included. S24

S17 The CASSCF active natural orbitals of the $[\mathbf{U^{Me_6}}]^-$, HS complex from the (10*e*, 13*o*) active space. An isosurface value of 0.04 a.u. was used. Occupation numbers and atomic contributions to the orbitals are included. S25

S18 The CASSCF active natural orbitals of the $[\mathbf{U^{Me_6}}]^-$, HS complex from the (10*e*, 18*o*) active space. An isosurface value of 0.04 a.u. was used. Occupation numbers and atomic contributions to the orbitals are included. S25

S19 The CASSCF active natural orbitals of the [$\mathbf{U}^{\mathbf{Me_6}}$]$^-$, IS complex from the (10*e*, 13*o*) active space. An isosurface value of 0.04 a.u. was used. Occupation numbers and atomic contributions to the orbitals are included. S26
S20 The CASSCF active natural orbitals of the [$\mathbf{U}^{\mathbf{Me_6}}$]$^-$, IS complex from the (10*e*, 18*o*) active space. An isosurface value of 0.04 a.u. was used. Occupation numbers and atomic contributions to the orbitals are included. S26

Molecular Geometries

Table S1: Comparison of the functional dependence of computed U−C and U−B bond distances in Å for the $\mathbf{U^{Me_6}}$ complex with respect to experiment.[1]

	U−C	U−B
Expt.[1]	2.932	2.568
PBE	2.826	2.512
PBE0	2.903	2.525
PBE0-D3	2.880	2.522
TPSS	2.837	2.521
TPSSh	2.869	2.526
TPSSh-D3	2.835	2.518
M06	3.024	2.556

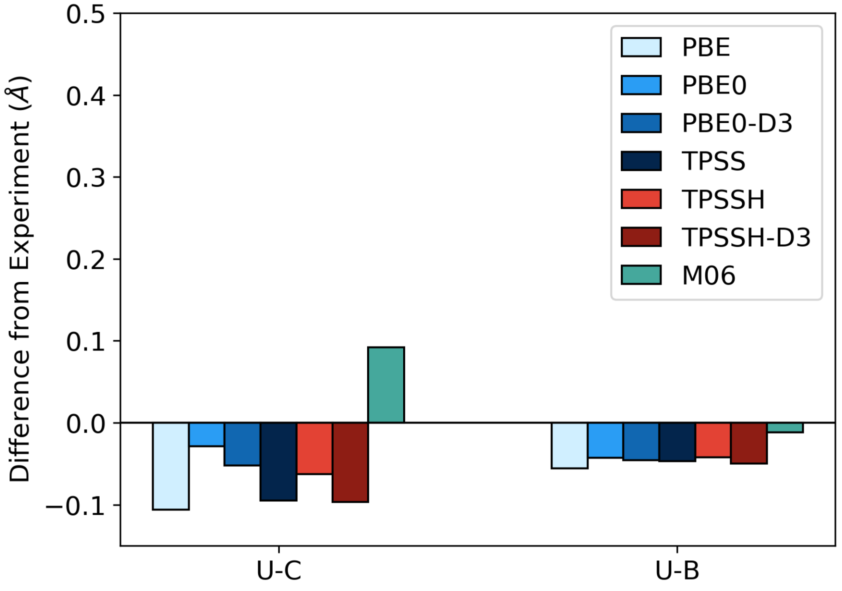

Figure S1: Comparison of the functional dependence of computed U−C and U−B bond distances in Å for the geometries of the $\mathbf{U^{Me_6}}$ complex with respect to experiment.[1]

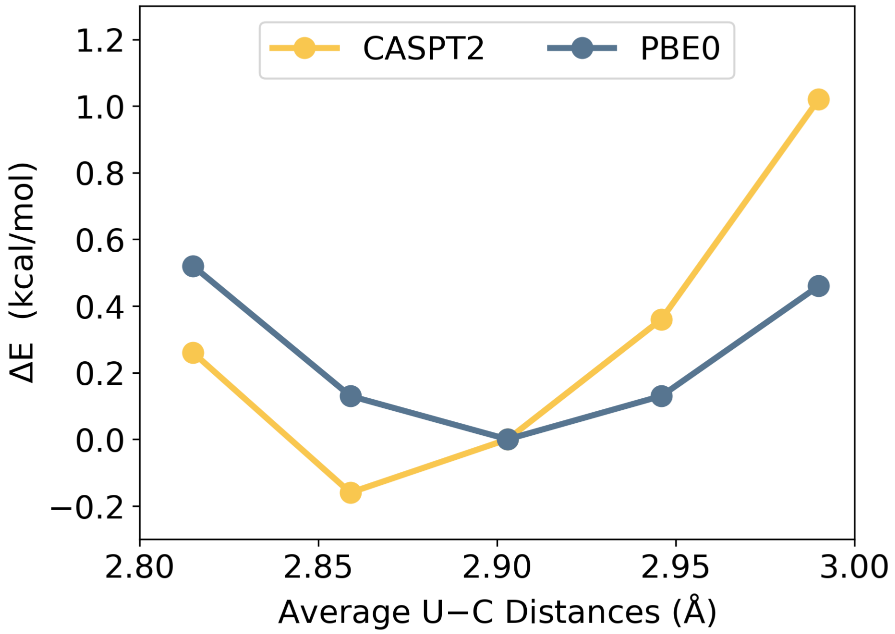

Figure S2: Potential energy surface in kcal/mol of the $\mathbf{U^{Me_6}}$ complex, computed with PBE0 and CASPT2, along the average U−C bond distance. The relative energy (ΔE) is given with reference to the energy on the PBE0 optimized geometry computed with each of the methods.

Topology Analysis

To understand the chemical bonding between uranium and arene moieties, we used the quantum theory of atoms in molecules (QTAIM) developed by Bader.[2] According to this theory, a chemical bond exists if there is a line of locally maximum electron density that links two neighboring atoms and a bond critical point (BCP) is identified. A BCP is defined as a minimum in the density along the locally maximal line. At a BCP, the gradient of the electron density ($\nabla\rho$) is zero, and the Laplacian ($\nabla^2\rho$) has either a net positive or net negative value. In the most clear cases, a positive Laplacian means a local depletion of charge, consistent with an ionic bond. On the other hand, a negative Laplacian indicates a local concentration of charge, which is a strong indication of a covalent bond. However, if the Laplacian is slightly positive, the total electronic energy density, E(r), which is the sum total of the kinetic and potential energy densities at the BCP, can be used to further classify the bond.[3] In a perfectly covalent bond, both the $\nabla^2\rho$ and E(r) are negative. A positive $\nabla^2\rho$ and a negative E(r) indicates a dative bond, a zero or close to zero E(r) indicates a metallic bond, and a positive E(r) indicates either an ionic or van der Waals bond. In all the systems we find positive $\nabla^2\rho$ and negative E(r) supporting the assignment of uranium-arene bonds as dative bonds.

We also report the delocalisation indices (δ) between the uranium and carbon atoms (Table S5 and S7) using the basin analysis approach as implemented in the Multiwfn 3.8 program.[4] This metric has been used in actinide chemistry to support the presence of degeneracy-driven covalency.

Table S2: The average topological properties of U−C bonds computed at the bond critical points (BCPs). All values are expressed in atomic units.

Acronym	ρ	$\nabla^2\rho$	G(r)	V(r)	E(r)
U^{Bz}	0.0327	0.0835	0.0238	-0.0269	-0.0030
U^{Tol}	0.0323	0.0827	0.0236	-0.0266	-0.0029
U^{Me_3}	0.0313	0.0792	0.0226	-0.0255	-0.0028
U^{Et_3}	0.0332	0.0815	0.0236	-0.0268	-0.0032
U^{iPr_3}	0.0315	0.0795	0.0226	-0.0255	-0.0028
U^{tBu_3}	0.0292	0.0776	0.0215	-0.0236	-0.0021
U^{Ph_3}	0.0313	0.0769	0.0221	-0.0249	-0.0028
U^{Me_6}	0.0300	0.0744	0.0212	-0.0238	-0.0026
$U^{TriPhen-T}$	0.0321	0.0865	0.0243	-0.0270	-0.0027
$[U(COT)_2]$	0.0474	0.113	0.0359	-0.0434	-0.0075

Table S3: The average topological properties of U−B bonds of the U^{Me_6} complex, computed at the bond critical points (BCPs). All values are expressed in atomic units.

Average U−B Distance (Å)	ρ	$\nabla^2\rho$	G(r)	V(r)	E(r)
2.525	0.0543	0.128	0.0451	-0.0582	-0.0132

Table S4: The PBE0-D3 average U−C distance of the $U^{TriPhen-C}$ complex together with the average topological properties of the U−C bonds computed at the bond critical points (BCPs). All values are expressed in atomic units.

Average U−C Distance (Å)	ρ	$\nabla^2\rho$	G(r)	V(r)	E(r)
3.009	0.0228	0.0636	0.0167	-0.0175	-0.0008

Table S5: Delocalization indices (δ) between the uranium and arene carbon atoms of all the $\mathbf{U^{arene}}$ complexes.

Complex (Å)	U-C1	U-C2	U-C3	U-C4	U-C5	U-C6	Average
U^{Me_6}	0.1575	0.1594	0.1529	0.1632	0.1519	0.1598	0.15745
U^{Bz}	0.1924	0.1923	0.1951	0.1901	0.1953	0.1967	0.19365
U^{Tol}	0.2012	0.1890	0.1656	0.1930	0.1976	0.1870	0.18890
U^{Me_3}	0.1697	0.1845	0.1711	0.1855	0.1705	0.1865	0.17796
U^{Et_3}	0.1724	0.1844	0.1726	0.1885	0.1742	0.1864	0.17975
U^{iPr_3}	0.1677	0.1772	0.1664	0.1830	0.1647	0.1795	0.17308
U^{tBu_3}	0.1503	0.1590	0.1697	0.1730	0.1401	0.1646	0.15945
U^{Ph_3}	0.1680	0.1799	0.1596	0.1828	0.1592	0.1771	0.17110
$U^{TriPhen-T}$	0.1884	0.1778	0.2050	0.1352	0.1315	0.2118	0.17495

Table S6: Delocalization indices (δ) between the uranium and boron atoms of all the **U^{arene}** complexes.

Complex (Å)	U-B1	U-B2	U-B3	Average
U^{Me_6}	0.1579	0.1575	0.1577	0.15770
U^{Bz}	0.1664	0.1608	0.1691	0.16543
U^{Tol}	0.1629	0.1633	0.1628	0.16300
U^{Me_3}	0.1621	0.1600	0.1612	0.16110
U^{Et_3}	0.1642	0.1604	0.1577	0.16076
U^{iPr_3}	0.1623	0.1585	0.1629	0.16123
U^{tBu_3}	0.1610	0.1568	0.1547	0.15750
U^{Ph_3}	0.1663	0.1707	0.1561	0.16436
$U^{TriPhen-T}$	0.1653	0.1666	0.1630	0.16496

Table S7: Delocalization indices (δ) between uranium and the carbon atoms of the $[U(COT)_2]$ complex.

Bonded Atoms	δ
U-C1	0.256
U-C2	0.252
U-C3	0.258
U-C4	0.273
U-C5	0.259
U-C6	0.249
U-C7	0.258
U-C8	0.273
U-C9	0.254
U-C10	0.263
U-C11	0.267
U-C12	0.256
U-C13	0.251
U-C14	0.262
U-C15	0.268
U-C16	0.256
Average	0.259

Energy Decomposition Analysis

To further characterize the bonding between the uranium and arene entities, we performed an energy decomposition analysis (EDA) by considering each complex as two fragments. One fragment corresponds to $U(BH_4)_3$ and the other moiety is the arene. Both fragments are neutral. The $U(BH_4)_3$ and arene fragments were computed for the quartet and singlet states, respectively. This allows one to evaluate the interaction energy between the two fragments of a molecule involved in bonding and to gain insight into the nature and strength of the interaction between the different fragments of a molecular system and how they contribute to the overall stability or reactivity of the complex. The interaction energy (ΔE_{Int}) is the sum total of the attractive and repulsive energies. When a calculation is performed without a dispersion correction, the following equation is used.

$$\Delta E_{Int} = \Delta E_{Pauli} + \Delta V_{Elstat} + \Delta E_{Orb} \quad (1)$$

The attractive terms have a negative sign and contain the electrostatic (ΔV_{Elstat}) and orbitallic (ΔE_{Orb}) energies, whereas the repulsive interaction solely consist of the Pauli repulsion energy (ΔE_{Pauli}) and are positive values. A negative value ΔE_{Int} supports dominance of the attractive interactions, thereby a stable chemical bond between the two fragments. In other words, the magnitude of the ΔE_{Int} corresponds to the energy required to dissociate the molecule into the two fragments (without including relaxation energy associated with geometric changes). Hence, the more negative the ΔE_{Int} value, the stronger the bonding between the two fragements. The interaction energies obtained by EDA for all of the complexes are summarized in Table S8.

Although we observed that the inclusion of the dispersion correction leads to U−C carbon distances with a larger difference from experiment in $\mathbf{U^{Me6}}$, it plays a significant role in evaluating the interaction energy in the energy decomposition analysis. In addition to electrostatic and orbitallic energies, the dispersion interaction acts as an additional attractive energy. In the presence of dispersion, the expression for the interaction energy can be decomposed using the following expression with the specific values for the complexes studied herein reported in Table S9.

$$\Delta E_{Int} = \Delta E_{Pauli} + \Delta V_{Elstat} + \Delta E_{Orb} + \Delta E_{Disp} \quad (2)$$

Table S8: Energy decomposition analysis of the uranium-arene complexes. All energies are reported in kcal/mol. The percent contribution of electrostatic and orbitallic interactions to the total attractive interaction energy is given in the parentheses.

Complex	ΔE_{Int}	ΔE_{Pauli}	$\Delta V_{Elstat.}$	$\Delta E_{Orb.}$
$\mathbf{U^{Me_6}}$	-39.6	77.2	-56.4 (48.3%)	-60.4 (51.7%)
$\mathbf{U^{Bz}}$	-34.6	78.8	-48.5 (42.7%)	-64.9 (57.2%)
$\mathbf{U^{Tol}}$	-36.1	78.9	-50.5 (43.9%)	-64.5 (56.1%)
$\mathbf{U^{Me_3}}$	-38.3	78.9	-53.8 (45.9%)	-63.4 (54.1%)
$\mathbf{U^{Et_3}}$	-38.0	83.3	-55.4 (45.6%)	-65.9 (54.3%)
$\mathbf{U^{^iPr_3}}$	-38.9	81.9	-56.2 (46.5%)	-64.6 (53.5%)
$\mathbf{U^{^tBu_3}}$	-38.6	78.5	-54.6 (46.6%)	-62.5 (53.3%)
$\mathbf{U^{Ph_3}}$	-35.3	77.9	-47.4 (42.1%)	-65.8 (58.1%)
$\mathbf{U^{TriPhen-T}}$	-34.9	74.0	-44.8 (41.1%)	-64.1 (58.8%)
$\mathbf{U^{TriPhen-C}}$	-22.2	45.6	-26.9 (39.6%)	-40.9 (60.3%)

Table S9: Energy decomposition analysis of the uranium-arene complexes considering the dispersion interaction between the two fragments. All energies are reported in kcal/mol. The percent contribution of electrostatic, orbital, and dispersion interactions to the total attractive interaction energy is given in the parenthesis.

Complex	ΔE_{Int}	ΔE_{Pauli}	$\Delta V_{Elstat.}$	$\Delta E_{Orb.}$	$\Delta E_{Disp.}$
$\mathbf{U^{Me_6}}$	-48.7	77.2	-56.4 (44.8%)	-60.4 (47.9%)	-9.1 (7.2%)
$\mathbf{U^{Bz}}$	-39.7	78.8	-48.5 (40.9%)	-64.9 (54.8%)	-5.1 (4.3%)
$\mathbf{U^{Tol}}$	-41.9	78.9	-50.5 (41.8%)	-64.5 (53.4%)	-5.8 (4.8%)
$\mathbf{U^{Me_3}}$	-45.5	78.9	-53.8 (43.2%)	-63.4 (50.9%)	-7.2 (5.8%)
$\mathbf{U^{Et_3}}$	-45.3	83.3	-55.4 (43.1%)	-65.9 (51.2%)	-7.3 (5.7%)
$\mathbf{U^{^iPr_3}}$	-48.1	81.9	-56.2 (43.2%)	-64.6 (49.7%)	-9.2 (7.1%)
$\mathbf{U^{^tBu_3}}$	-49.6	78.5	-54.6 (42.6%)	-62.5 (48.8%)	-11.0 (8.6%)
$\mathbf{U^{Ph_3}}$	-44.7	77.9	-47.4 (38.7%)	-65.8 (53.7%)	-9.4 (7.6%)
$\mathbf{U^{TriPhen-T}}$	-42.4	74.0	-44.8 (38.5%)	-64.1 (55.1%)	-7.5 (6.4%)
$\mathbf{U^{TriPhen-C}}$	-31.1	45.6	-26.9 (35.1%)	-40.9 (53.3%)	-8.9 (11.6%)

To assess the impact of dispersion on ΔE_{Int}, we performed the energy decomposition analysis using the PBE0-D3 functional. We considered the previously computed PBE0 geometries and divided them into two fragments, $U(BH_4)_3$ and arene, and then calculated the ΔE_{Int}. Our computations show that the magnitudes of ΔV_{Elstat} and ΔE_{Orb} remained the same; however, the presence

of dispersion contributed attractive energies ranging from approximately 5 to 11 kcal/mol across the complexes (Table S9) resulting in a ΔE_{Int} thatw as more negative by that amount. Furthermore, the presence of this additional attractive energy reduced the percentage contribution of ΔV_{Elstat} and ΔE_{Orb}, compared to the values observed previously. However, the conclusion regarding the nature of the bond remains unchanged.

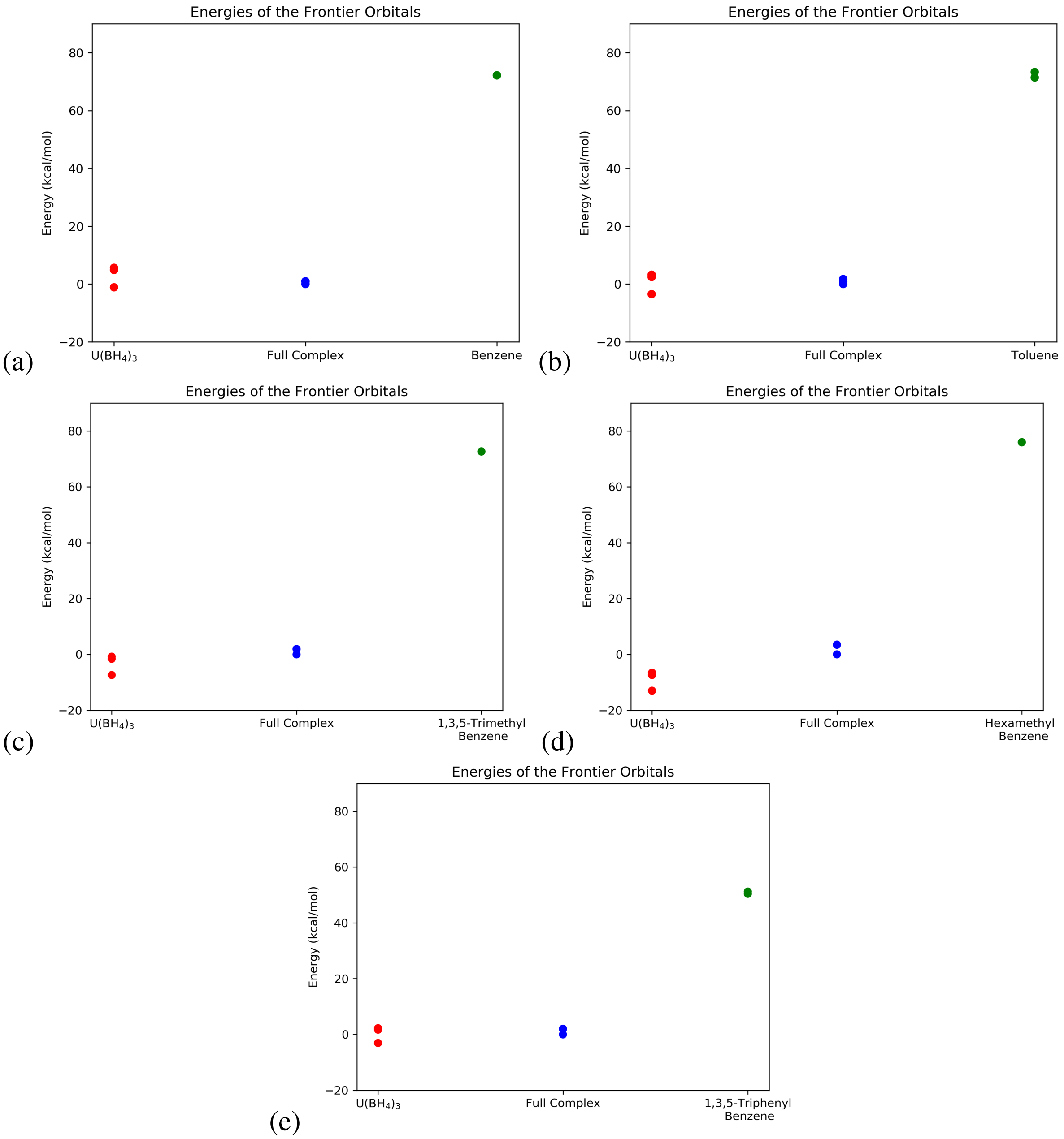

Figure S3: Frontier orbital energies in kcal/mol of the two fragments and the total complex for (a) $\mathbf{U^{Bz}}$, (b) $\mathbf{U^{Tol}}$, (c) $\mathbf{U^{Me_3}}$, (d) $\mathbf{U^{Me_6}}$, and (e) $\mathbf{U^{Ph_3}}$.

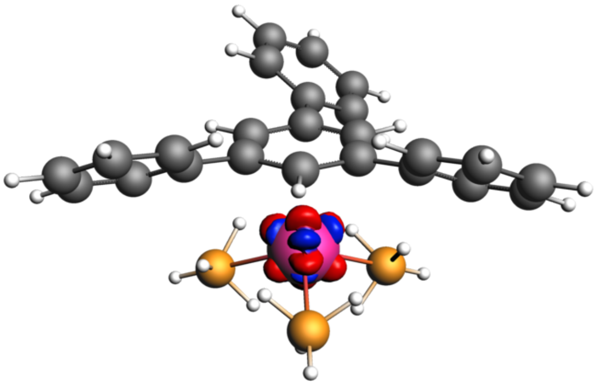

Figure S4: The ETS-NOCV deformation density of the $\mathbf{U^{Ph_3}}$ with isosurface value of 0.01 a.u., as recommended in the ADF manual. The associated $\alpha - \Delta\rho$ ΔE = -10.465 kcal/mol. No surface is visible at the lower $\alpha - \Delta\rho$ ΔE or $\beta - \Delta\rho$ ΔE values.

CASSCF Analysis

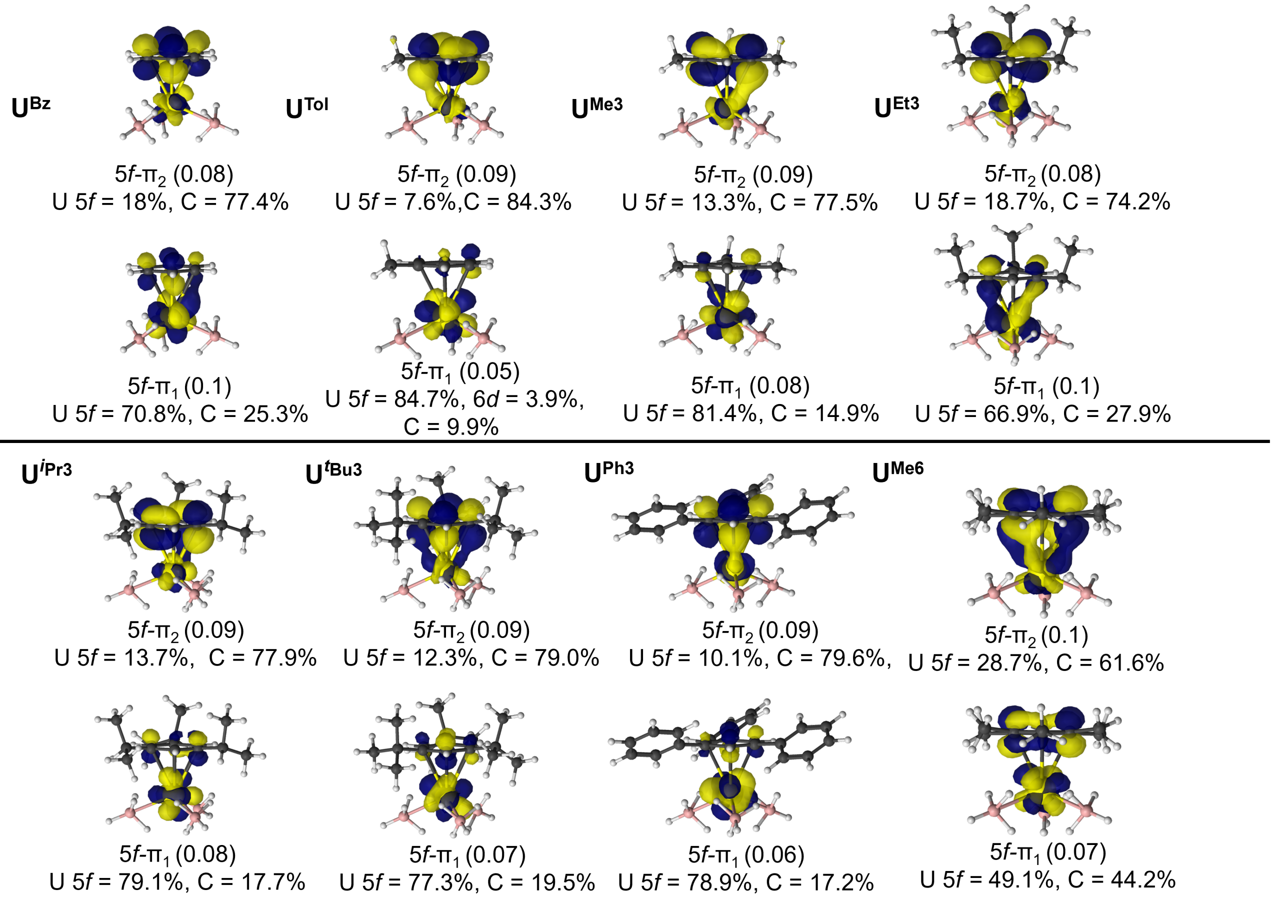

Figure S5: CASSCF active natural orbitals with dominant mixing between the metal $5f$ and the ligand π orbitals (labeled $5f$-π_n) of the eight complexes are shown. The full active space orbitals are shown in the Figures S8 to S20. An isovalue of 0.04 a.u is used.

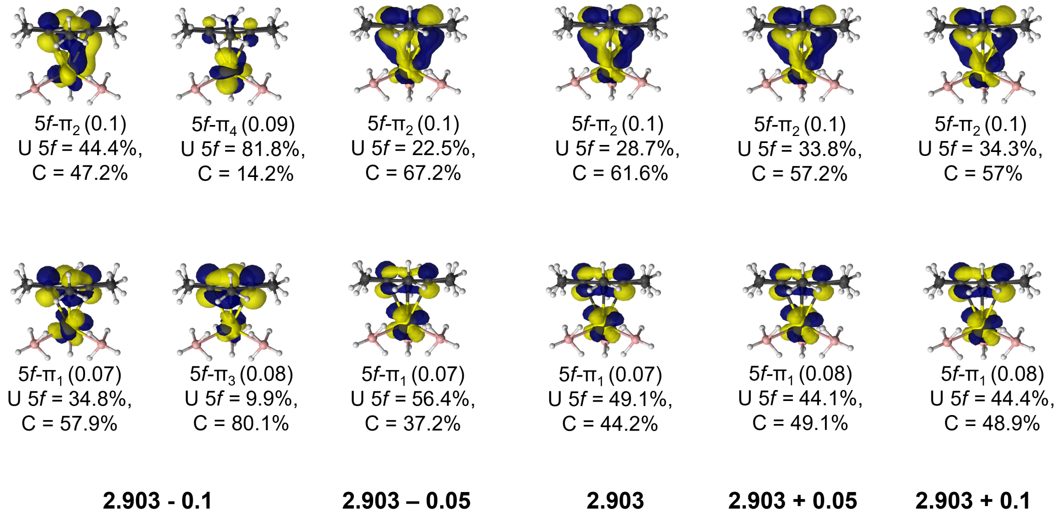

Figure S6: The CASSCF active natural orbitals of the **U^{Me_6}** complex with significant metal-ligand overlap are shown with respect to the average uranium-arene distances. The deviation from the equilibrium uranium-arene distances in Å are reported underneath of the orbitals.

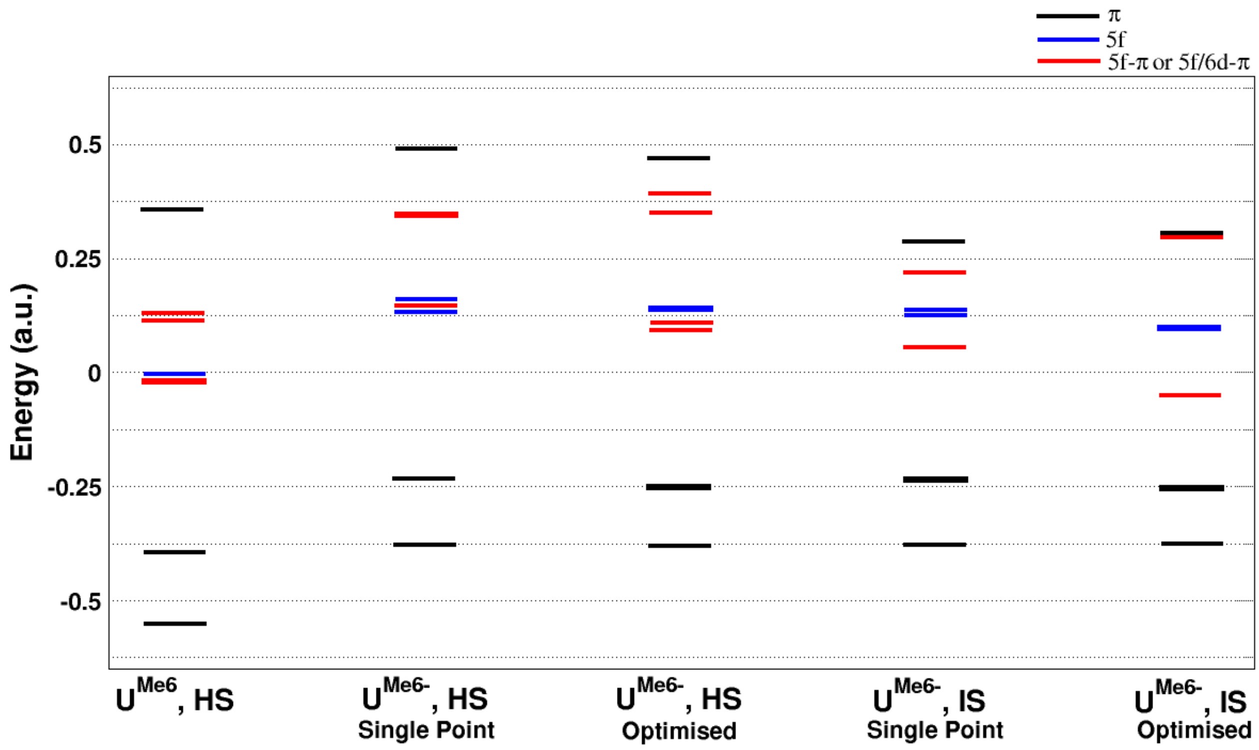

Figure S7: Canonical orbital energy diagram from the 18 orbital active space calculations.

Table S10: Canonical orbital energies (in a.u.) from the 18 orbital active space calculations.

$\mathbf{U^{Me_6}}$	$[\mathbf{U^{Me_6}}]^-$, HS Single Point	$[\mathbf{U^{Me_6}}]^-$, HS Optimised	$[\mathbf{U^{Me_6}}]^-$, IS Single Point	$[\mathbf{U^{Me_6}}]^-$, IS Optimised
-0.5508	-0.38	-0.381	-0.3784	-0.3771
-0.3948	-0.2345	-0.2545	-0.2357	-0.2578
-0.3944	-0.2344	-0.2503	-0.2316	-0.2516
-0.0215	0.1351	0.0965	0.0575	-0.0486
-0.02	0.1419	0.1136	0.1311	0.0976
-0.0132	0.1426	0.1233	0.1384	0.1021
0.1174	0.1451	0.1301	0.2231	0.2991
0.1289	0.3455	0.3493	0.2881	0.3034
0.3173	0.3488	0.3959	0.4707	0.4174
0.3227	0.475	0.4669	0.4807	0.4527
0.3559	0.4756	0.4757	0.4974	0.55
0.4332	0.4919	0.4911	0.598	0.6289
0.547	0.5847	0.6227	0.7184	0.7288
0.7047	0.7088	0.6996	0.7625	0.7921
0.8455	0.7251	0.7437	0.8799	0.8779
0.8912	0.7267	0.7662	0.9267	0.9167
0.9712	0.8894	0.8749	0.9394	1.0065
1.0126	0.927	0.8945	1.0543	1.0329

Table S11: Mulliken spin population analysis from CASSCF-(9*e*,18*o*) for the high-spin quartet state of $\mathbf{U^{Me_6}}$, and with (10*e*,18*o*) active space for the high-spin quintet state of $[\mathbf{U^{Me_6}}]^-$, and the intermediate-spin triplet state of $[\mathbf{U^{Me_6}}]^-$.

Complex	U	C1	C2	C3	C4	C5	C6	$\sum_{\text{Arene}}$
U^{Me_6}, HS	2.9436	0.0082	0.0066	0.0091	0.0001	0.0007	0.0076	0.0323
$[U^{Me_6}]^-$, HS	3.0681	0.1397	0.1029	0.1995	0.1164	0.1253	0.1770	0.8608
$[U^{Me_6}]^-$, IS	2.2110	-0.0050	-0.0875	-0.0154	-0.0156	-0.0831	-0.0079	-0.2145

Table S12: LoProp charge analysis from CASSCF-(9*e*,18*o*) for the high-spin quartet state of $\mathbf{U^{Me_6}}$, and with (10*e*,18*o*) active space for the high-spin quintet state of $[\mathbf{U^{Me_6}}]^-$, and the intermediate-spin triplet state of $[\mathbf{U^{Me_6}}]^-$.

Complex	U	C1	C2	C3	C4	C5	C6	$\sum_{\text{Arene}}$
U^{Me_6}, HS	2.4513	-0.0213	-0.0437	-0.0224	-0.0389	-0.0182	-0.0467	-0.1912
$[U^{Me_6}]^-$, HS	2.4168	-0.1081	-0.1177	-0.1403	-0.1262	-0.0998	-0.1606	-0.7527
$[U^{Me_6}]^-$, IS	2.5849	-0.0617	-0.3086	-0.0607	-0.0729	-0.3115	-0.0827	-0.8981

Table S13: Absolute CASPT2 (9*e*,13*o*) and PBE0 energies (a.u.) of the ground quartet state of the $\mathbf{U}^{\mathbf{Me_6}}$ complex corresponding to the PES of Figure S2.

	2.903−0.1 Å	2.903−0.05 Å	2.903	2.903+0.05 Å	2.903+0.1 Å
E_{CASPT2}	-28498.603004739	-28498.6037151178	-28498.6034370608	-28498.6028527379	-28498.6017974557
E_{PBE0}	-1026.335778250	-1026.336400552	-1026.336608067	-1026.336394729	-1026.335871500

Table S14: Absolute CASPT2 energies (a.u.) corresponding to the PES of Figure 8a. An Active space of (9*e*,18*o*) was used for evaluating $E_{Quartet}$, and the $E_{Quintet}$, and $E_{Triplet}$ were computed using (10*e*,18*o*) active space.

	$\mathbf{U}^{\mathbf{Me_6}}$, HS	I1	I2	I3	I4	$[\mathbf{U}^{\mathbf{Me_6}}]^-$, HS
$E_{Quartet}$	-28498.5959714201	-28498.5954916165	-28498.5942204689	-28498.5934098285	-28498.5922961563	-28498.5897423481
$E_{Quintet}$	-28498.6262095252	-28498.6283884740	-28498.6307682094	-28498.6342281971	-28498.6380039561	-28498.6408903173
$E_{Triplet}$	-28498.6095694480	-28498.6123310884	-28498.6153750954	-28498.6195880627	-28498.6242876619	-28498.6282542741

Table S15: Absolute CASPT2 energies (a.u.) corresponding to the PES of Figure 8b. An Active space of (9*e*,18*o*) was used for evaluating $E_{Quartet}$, and the $E_{Quintet}$, and $E_{Triplet}$ were computed using (10*e*,18*o*) active space.

	$\mathbf{U}^{\mathbf{Me_6}}$, HS	I1	I2	I3	I4	$[\mathbf{U}^{\mathbf{Me_6}}]^-$, IS
$E_{Quartet}$	-28498.5959740642	-28498.5814977902	-28498.5644018224	-28498.5616196076	-28498.5721129534	-28498.5765255830
$E_{Quintet}$	-28498.6262029453	-28498.6127091675	-28498.5996640030	-28498.5981476710	-28498.6147633195	-28498.6252653975
$E_{Triplet}$	-28498.6095683761	-28498.6032829348	-28498.5951505379	-28498.6017182725	-28498.6216692147	-28498.6363624078

CASSCF Active Orbitals of U^{arene} Complexes

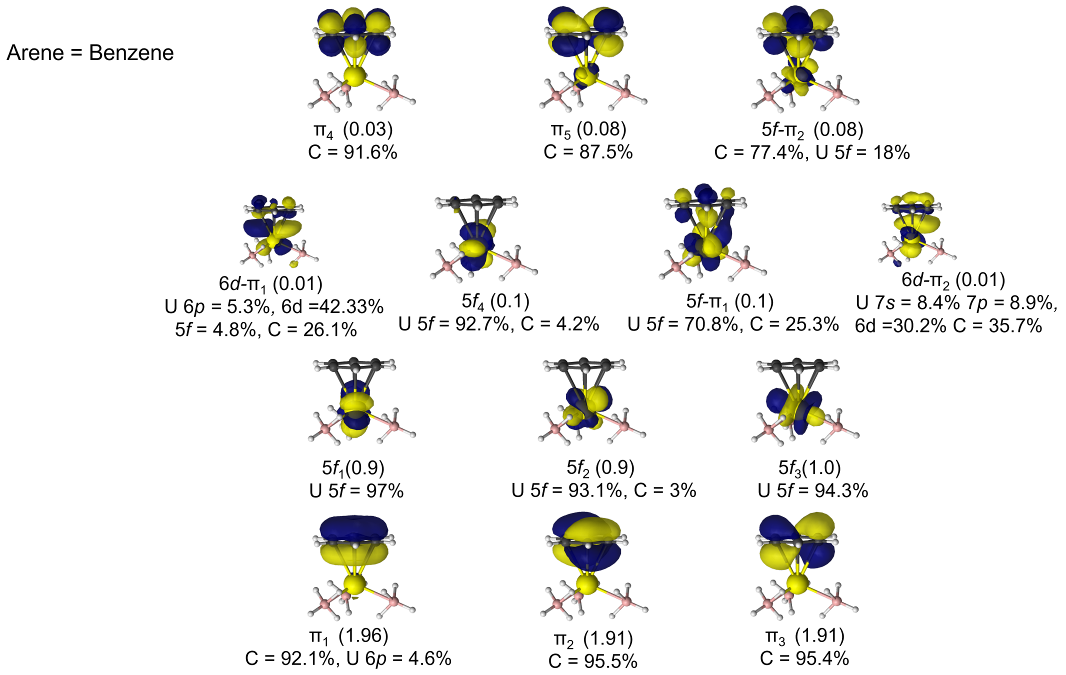

Figure S8: The CASSCF active natural orbitals of the $\mathbf{U^{Bz}}$ complex from the ($9e$, $13o$) active space. An isosurface value of 0.04 a.u. was used. Occupation numbers and atomic contributions to the orbitals are included.

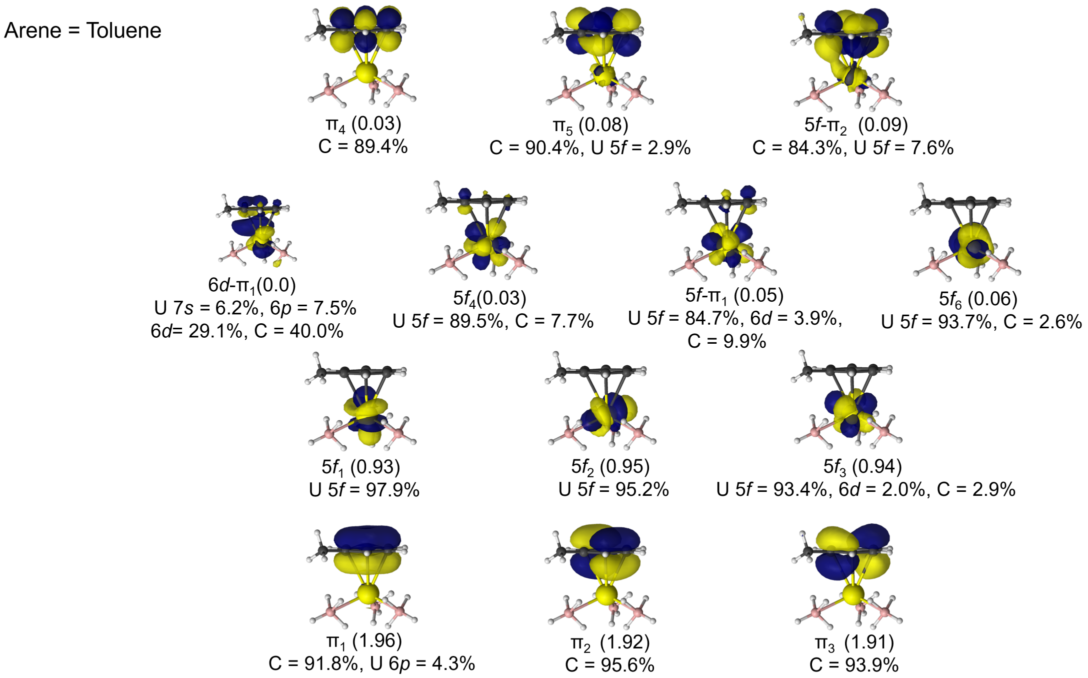

Figure S9: The CASSCF active natural orbitals of the $\mathbf{U^{Tol}}$ complex from the (9*e*, 13*o*) active space. An isosurface value of 0.04 a.u. was used. Occupation numbers and atomic contributions to the orbitals are included.

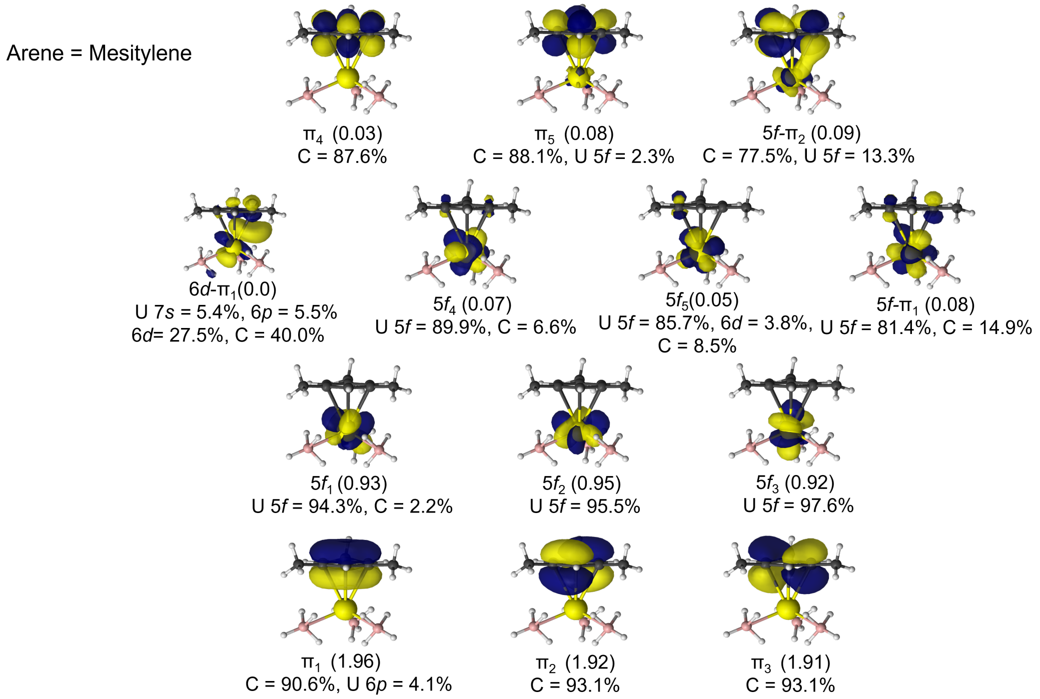

Figure S10: The CASSCF active natural orbitals of the $\mathbf{U^{Me_3}}$ complex from the (9*e*, 13*o*) active space. An isosurface value of 0.04 a.u. was used.

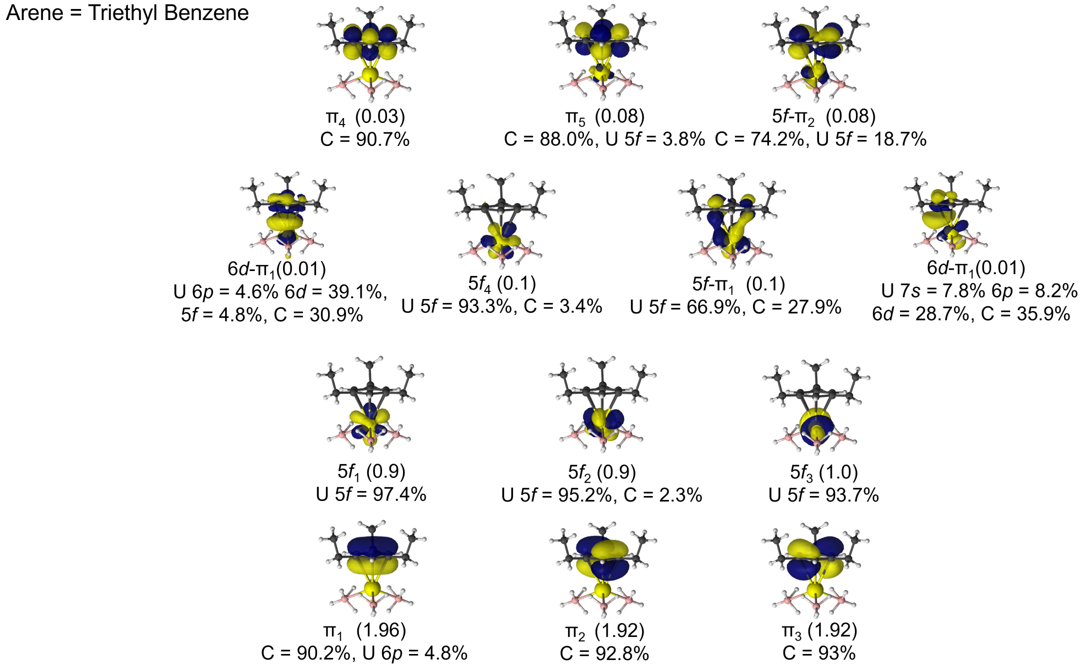

Figure S11: The CASSCF active natural orbitals of the $\mathbf{U^{Et_3}}$ complex from the (9*e*, 13*o*) active space. An isosurface value of 0.04 a.u. was used. Occupation numbers and atomic contributions to the orbitals are included.

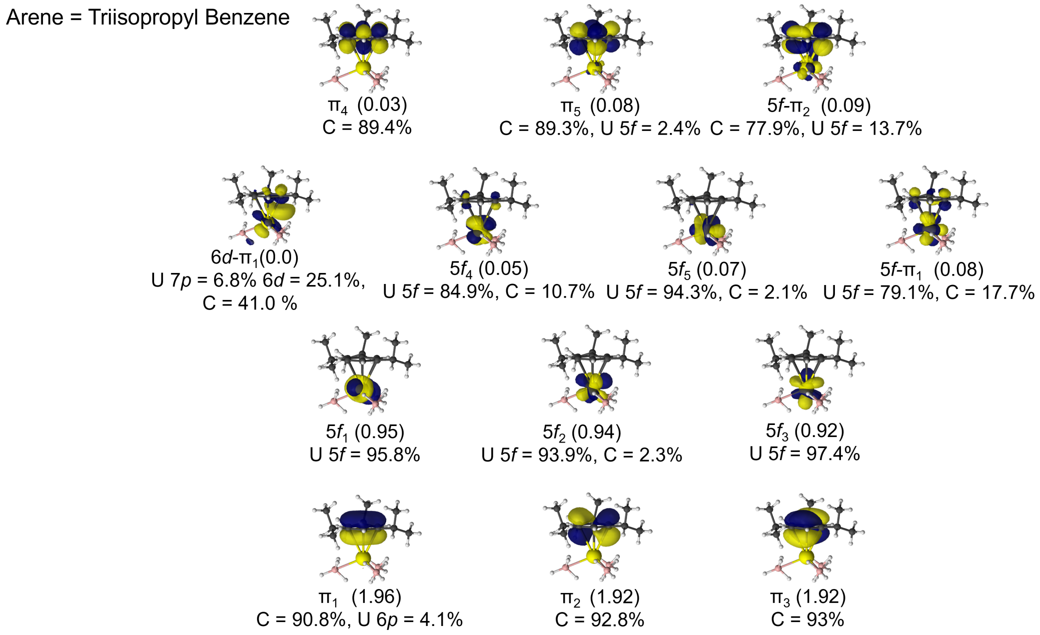

Figure S12: The CASSCF active natural orbitals of the $\mathbf{U^{iPr_3}}$ complex from the (9*e*, 13*o*) active space. An isosurface value of 0.04 a.u. was used. Occupation numbers and atomic contributions to the orbitals are included.

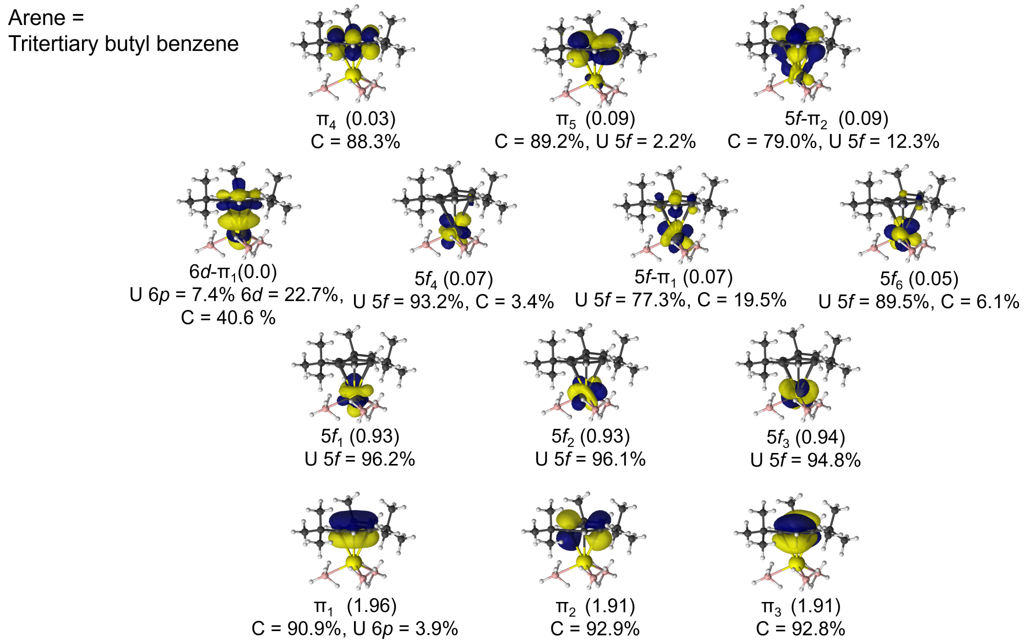

Figure S13: The CASSCF active natural orbitals of the $\mathbf{U^{tBu_3}}$ complex from the (9e, 13o) active space. An isosurface value of 0.04 a.u. was used. Occupation numbers and atomic contributions to the orbitals are included.

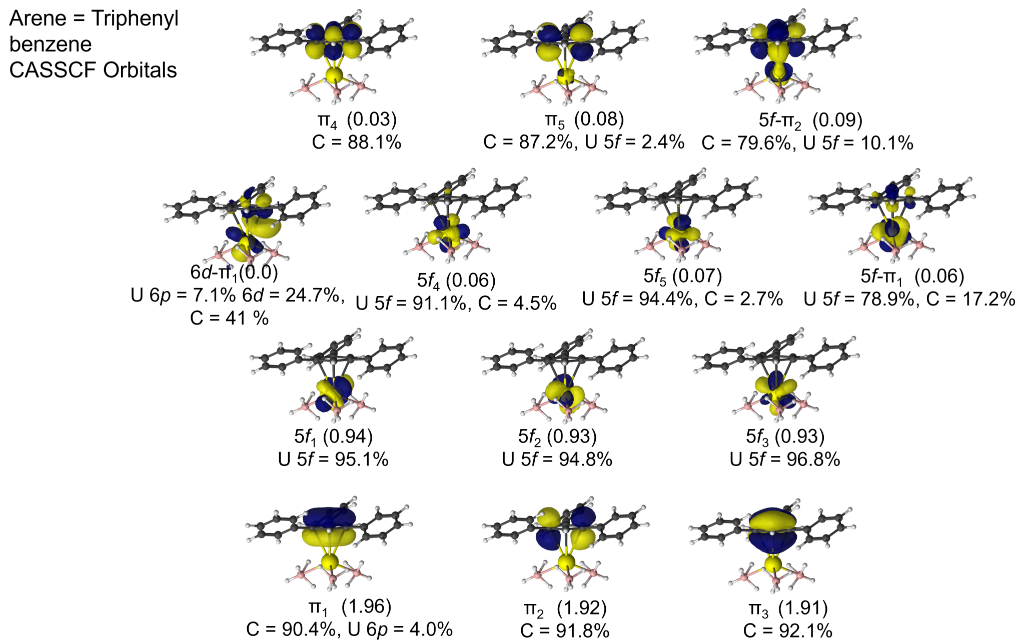

Figure S14: The CASSCF active natural orbitals of the $\mathbf{U^{Ph_3}}$ complex from the (9e, 13o) active space. An isosurface value of 0.04 a.u. was used. Occupation numbers and atomic contributions to the orbitals are included.

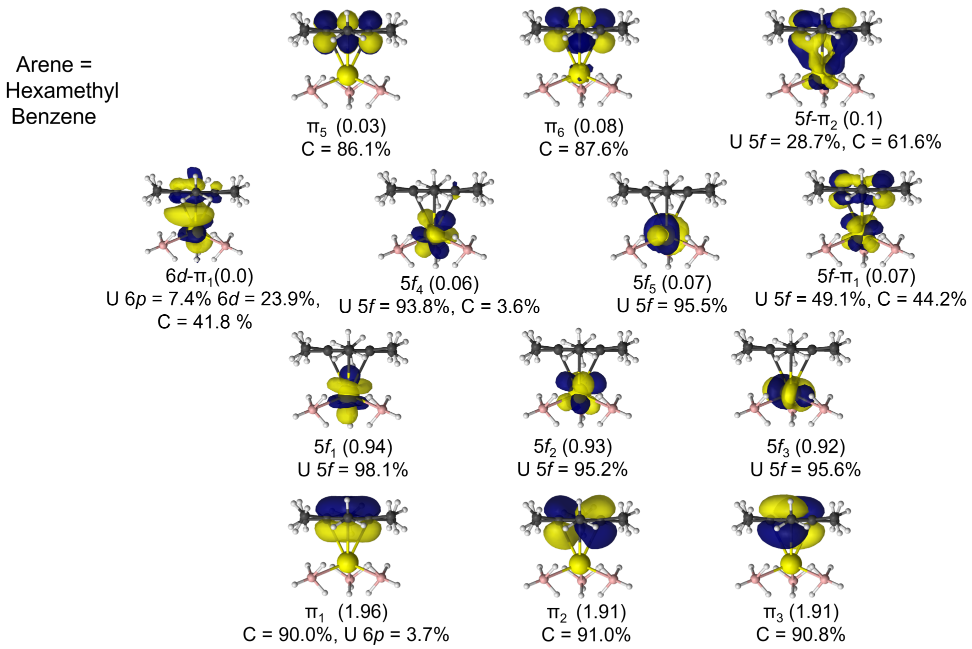

Figure S15: The CASSCF active natural orbitals of the $\mathbf{U}^{\mathbf{Me_6}}$ complex from the (9e, 13o) active space. An isosurface value of 0.04 a.u. was used. Occupation numbers and atomic contributions to the orbitals are included.

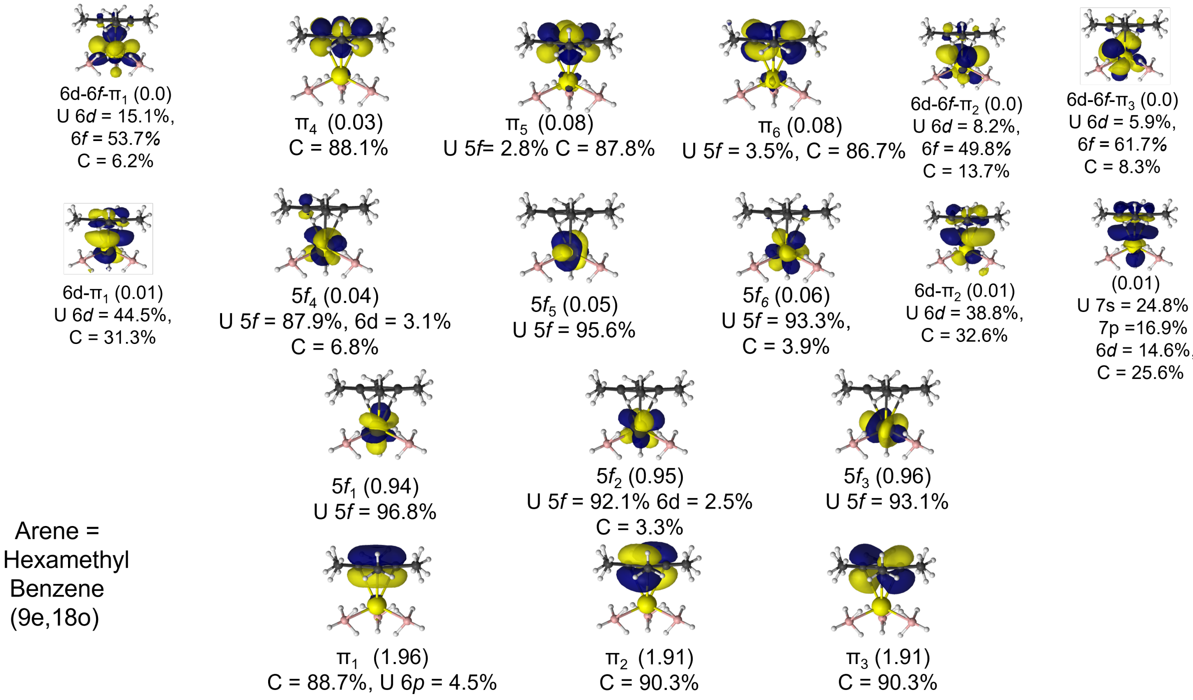

Figure S16: The CASSCF active natural orbitals of the $\mathbf{U}^{\mathbf{Me_6}}$ complex from the (9e, 18o) active space. An isosurface value of 0.04 a.u. was used. Occupation numbers and atomic contributions to the orbitals are included.

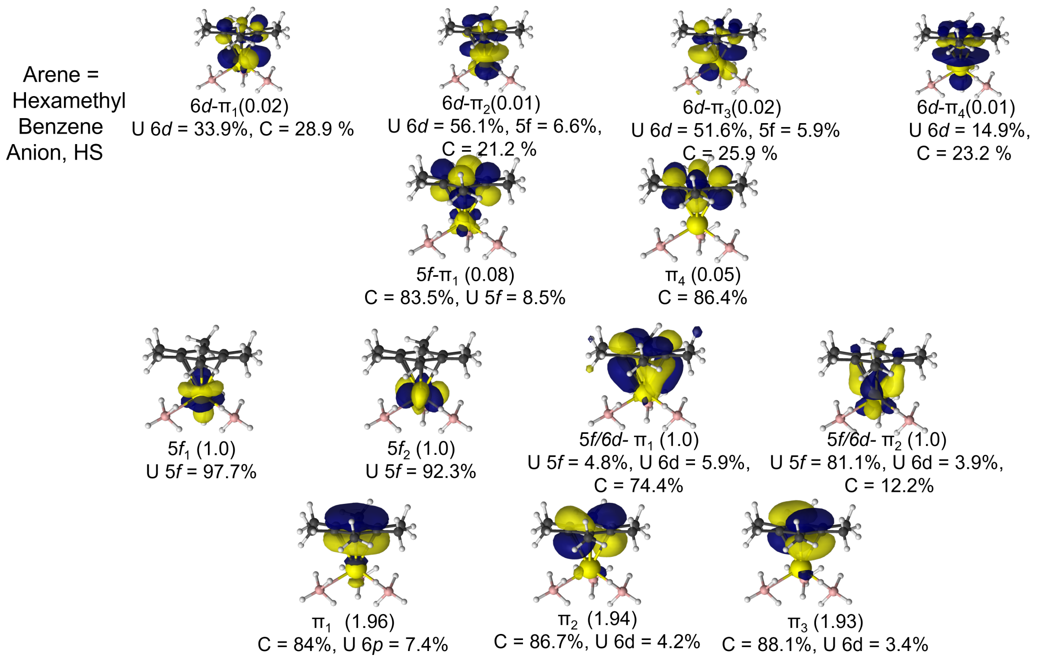

Figure S17: The CASSCF active natural orbitals of the $[\mathbf{U}^{\mathbf{Me_6}}]^-$, HS complex from the (10e, 13o) active space. An isosurface value of 0.04 a.u. was used. Occupation numbers and atomic contributions to the orbitals are included.

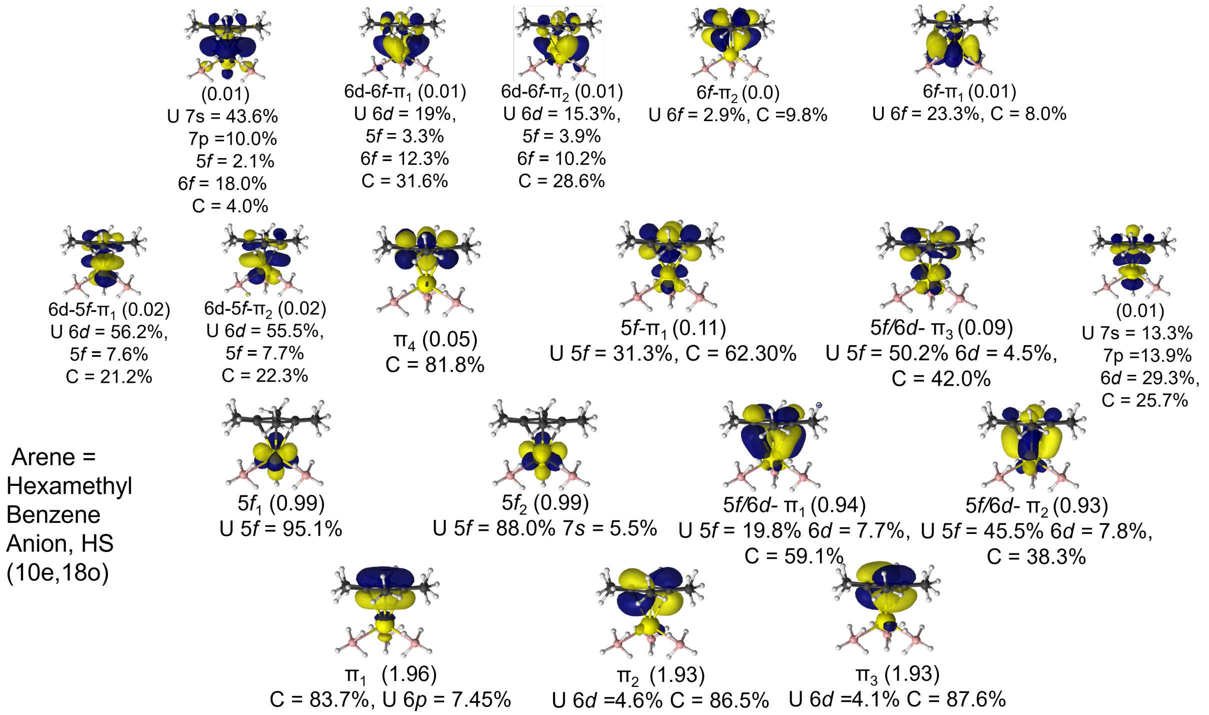

Figure S18: The CASSCF active natural orbitals of the $[\mathbf{U}^{\mathbf{Me_6}}]^-$, HS complex from the (10e, 18o) active space. An isosurface value of 0.04 a.u. was used. Occupation numbers and atomic contributions to the orbitals are included.

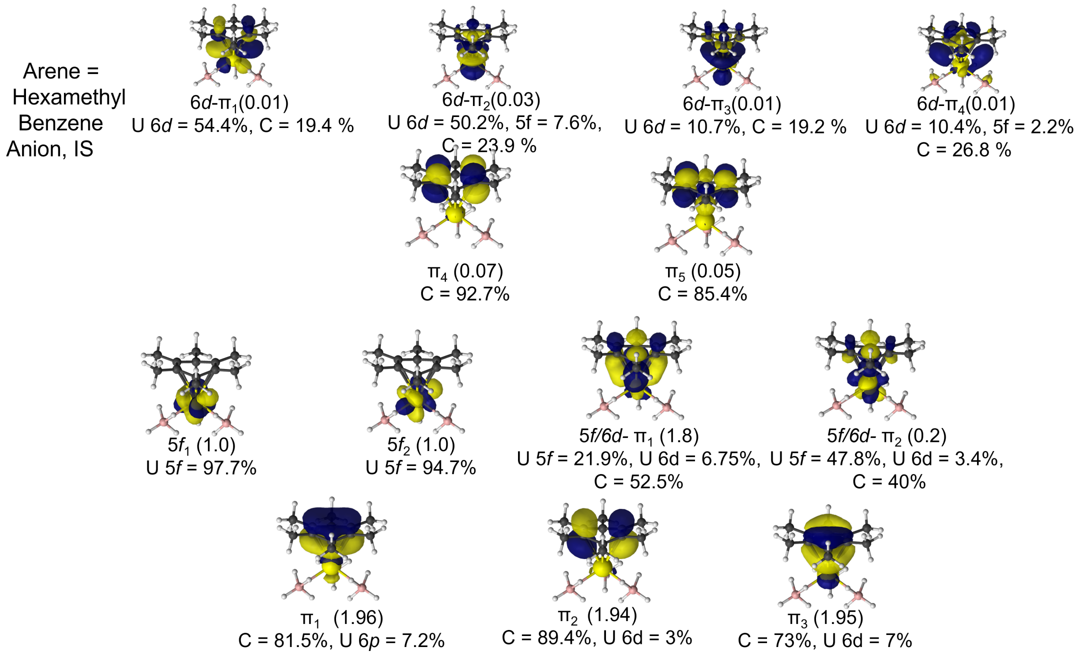

Figure S19: The CASSCF active natural orbitals of the $[\mathbf{U}^{\mathbf{Me_6}}]^-$, IS complex from the (10*e*, 13*o*) active space. An isosurface value of 0.04 a.u. was used. Occupation numbers and atomic contributions to the orbitals are included.

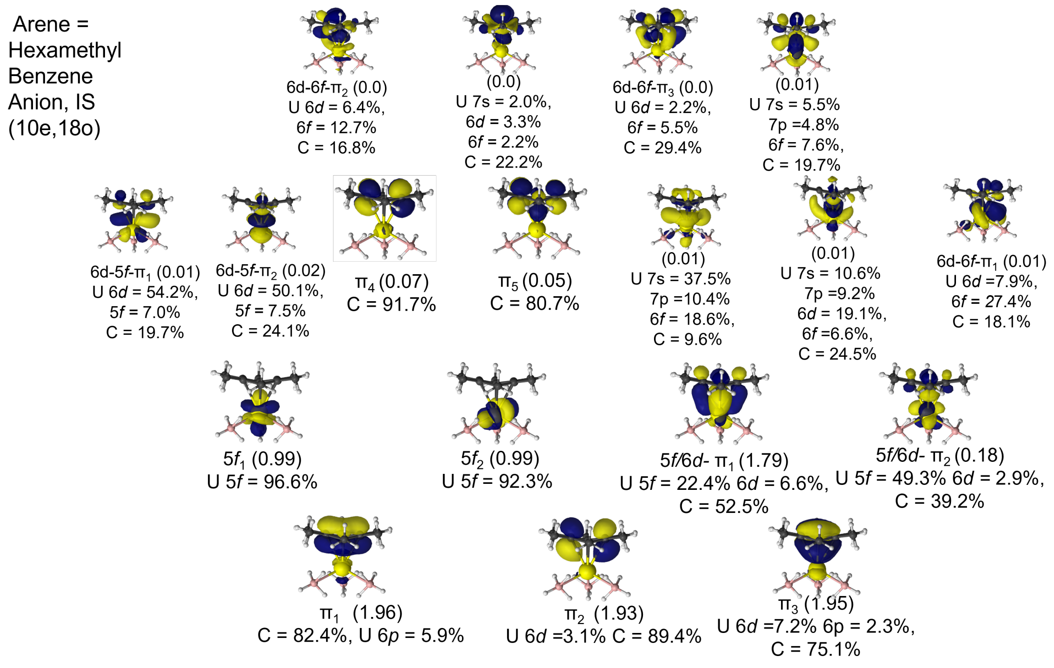

Figure S20: The CASSCF active natural orbitals of the $[\mathbf{U}^{\mathbf{Me_6}}]^-$, IS complex from the (10*e*, 18*o*) active space. An isosurface value of 0.04 a.u. was used. Occupation numbers and atomic contributions to the orbitals are included.

References

(1) Baudry, D.; Bulot, E.; Charpin, P.; Ephritikhine, M.; Lance, M.; Nierlich, M.; Vigner, J. Arene uranium borohydrides: synthesis and crystal structure of $(\eta - C_6Me_6)U(BH_4)_3$. *J. Organomet. Chem.* **1989**, *371*, 155–162.

(2) Bader, R. F. W. A Bond Path: A Universal Indicator of Bonded Interactions. *J. Phys. Chem. A* **1998**, *102*, 7314–7323.

(3) Bianchi, R.; Gervasio, G.; Marabello, D. Experimental Electron Density Analysis of $Mn_2(CO)_{10}$: MetalMetal and MetalLigand Bond Characterization. *Inorg. Chem.* **2000**, *39*, 2360–2366.

(4) Lu, T.; Chen, F. Multiwfn: A multifunctional wavefunction analyzer. *J. Comp. Chem.* **2012**, *33*, 580–592.
